# Stereodivergent synthesis of jaspine B and its isomers using a carbohydrate-derived alkoxyallene as C_3_-building block

**DOI:** 10.3762/bjoc.9.291

**Published:** 2013-11-19

**Authors:** Volker Martin Schmiedel, Stefano Stefani, Hans-Ulrich Reissig

**Affiliations:** 1 Institut für Chemie und Biochemie, Freie Universität Berlin, Takustr. 3, D-14195 Berlin, Germany

**Keywords:** chiral auxiliaries, gold catalysis, jaspine B, lithiated alkoxyallenes, natural product synthesis, pachastrissamine, tetrahydrofurans

## Abstract

Herein we present the synthesis of the anhydrophytosphingosine jaspine B and three of its stereoisomers using a carbohydrate-derived alkoxyallene in order to obtain the products in enantiopure form. Key step of the reaction sequence is the addition of the lithiated alkoxyallene to pentadecanal, setting the configuration at the later C-2 of the ring system. This reaction step proceeds with moderate selectivity and therefore leads to a stereodivergent approach to the natural product and its enantiomer. The gold-catalyzed 5-*endo*-cyclization affords the corresponding dihydrofurans, which after separation, azidation of the enol ether moiety and two subsequent reduction steps give the natural product and its stereoisomers.

## Introduction

**Scheme 1:**
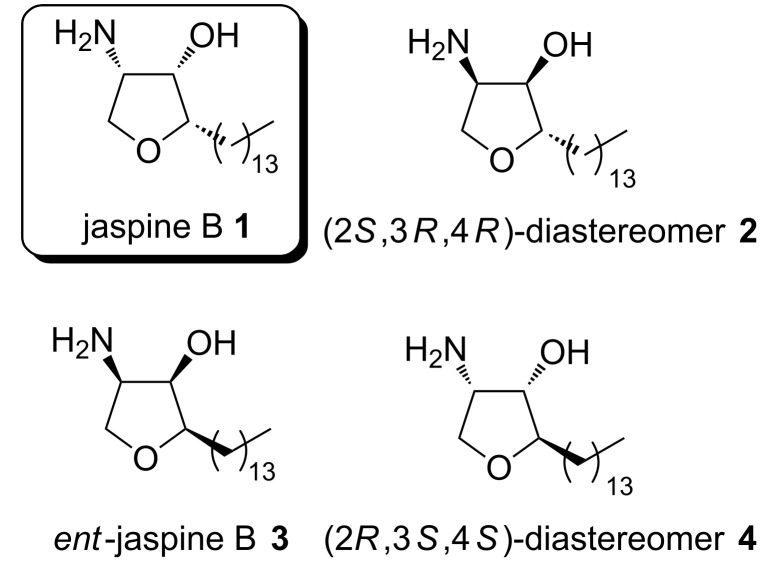
Structure of jaspine B **1** and its stereoisomers **2**–**4**.

Jaspine B, also known as pachastrissamine (**1**, Scheme 1), is an anhydrophytosphingosine derivative, isolated 2002 from the marine sponge *Pachastrissa sp.* by Higa et al. [1]. In 2003 Debitus et al. were able to extract this natural product from the marine sponge *Jaspis sp.* [2]. Jaspine B (**1**) comprises a densely functionalized tetrahydrofuran ring, bearing three contiguous (2*S,*3*S,*4*S*)-configured stereogenic centers with a long alkyl chain at C-2 and a 1,2-amino alcohol moiety at C-3 and C-4. Due to its interesting structure and biological activities, for instance cytotoxicity and apoptosis promotion of several cancer cell lines [3–6] the synthesis of this natural product attracted the attention of many research groups resulting in more than 30 published syntheses up to now. Most frequently they are based on transformations of starting materials available from the ”chiral pool”, e.g. L-serine [7–18], or by asymmetric catalysis [19–25]. Several publications also focused on the synthesis of stereoisomers of the natural product [4,12,16–17,19,26–28] because these compounds also showed comparable biological activities [4–5].

**Scheme 2:**
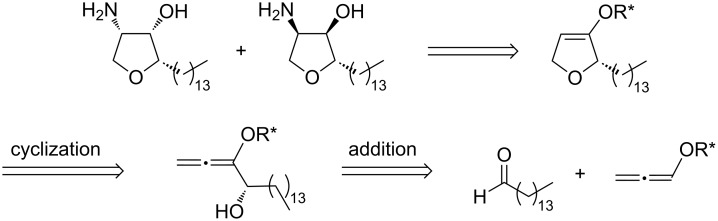
Retrosynthetic analysis of jaspine B leading to pentadecanal and an alkoxyallene.

During our current studies on the synthesis of highly functionalized heterocycles utilizing alkoxyallenes as versatile C_3_-building blocks we chose jaspine B (**1**) as an attractive target [29–33]. Our approach to **1** is based on the addition of a lithiated alkoxyallene with a chiral auxiliary to pentadecanal as electrophile. This step will generate the first stereogenic center and install the C_14_-alkyl chain at the later C-2 position of the natural product (Scheme 2). A subsequent 5-*endo*-cyclization of the resulting allenyl alcohols leads to a mixture of diastereomeric dihydrofurans, which can be separated by HPLC (only the (*S*)-enantiomer is depicted). This allows the synthesis of the natural product and its enantiomer by functionalization of both diastereomers. The *cis*-configured amino alcohol moiety at C-3 and C-4 is installed by azidation and subsequent reduction steps leading to the final products. The presented strategy allows the preparation of jaspine B, its enantiomer and two diastereomers, all of which are known for their biological activities (Scheme 2).

## Results and Discussion

### Synthesis of racemic jaspine B and its C-2-epimer

**Scheme 3:**
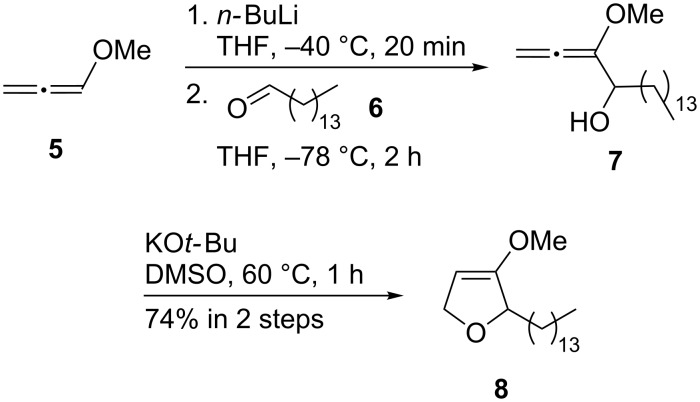
Synthesis of racemic dihydrofuran **8**.

Initial studies examined the feasibility of this planned approach for the generation of the functionalized tetrahydrofuran system. They were based on methoxyallene (**5**) and led to the preparation of the racemic natural product. Methoxyallene (**5**), which is easily prepared in two steps from propargyl alcohol in large scale [34], was deprotonated with *n-*BuLi and the generated lithiated methoxyallene was subsequently treated with pentadecanal (**6**). The resulting allenyl alcohol **7** was not sufficiently stable for purification and hence was directly used for the next cyclization step (Scheme 3). After a 5*-endo-*cyclization of **7** employing KO*t-*Bu in DMSO dihydrofuran derivative **8** was obtained in 74% overall yield from **5**. The strongly basic conditions as first described by Brandsma and Arens [35] used for the cyclization of **7** gave a higher yield than the attempted gold(I) catalysis (39% overall yield), which might be due to the deactivation of the gold catalyst by impurities still present in the crude allenyl alcohol **7**.

**Scheme 4:**
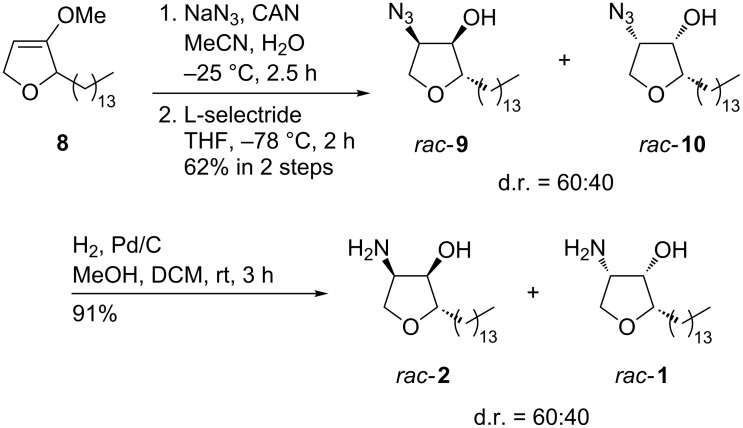
Synthesis of racemic jaspine B *rac*-**1** and its diastereomer *rac*-**2**.

Next, several methods for the introduction of the amino group were examined. We were very pleased to discover that the oxidative azidation [36] allowed a direct access to the corresponding α-azidofuranones in one step. The two diastereomers of the azides were obtained in a 60:40 ratio. The subsequent reduction of the carbonyl group with L-selectride in THF furnished the α-azidotetrahydrofuranols *rac*-**9** and *rac*-**10** in a satisfying yield of 62% over two steps (Scheme 4). Only the two diastereomers shown in Scheme 4 were isolated, whereas the two other possible isomers could not be detected. The obtained diastereoselectivity of the reduction is likely due to stereoelectronic effects of the α-azido group overcoming the steric hindrance of the slim alkyl chain. We assume that in both diastereomers the azido group occupies a pseudoaxial position shielding this face of the furanone ring and therefore steers the hydride attack to the other side. Similar observations have been reported for the reduction of carbonyl compounds bearing an electronegative group in α-position [37]. After palladium-catalyzed hydrogenolysis of α-azidotetrahydrofuranols *rac*-**9** and *rac*-**10** an inseparable mixture of racemic jaspine B (*rac*-**1**) and its C-2-epimer *rac*-**2** was obtained. Hence the natural product and its diastereomer were obtained in five steps with a good overall yield of 42% with respect to aldehyde **6**. This result was very encouraging and we therefore started the preparation of the enantiopure natural product and its stereoisomers.

### Synthesis of enantiopure jaspine B using a carbohydrate-derived alkoxyallene

**Scheme 5:**
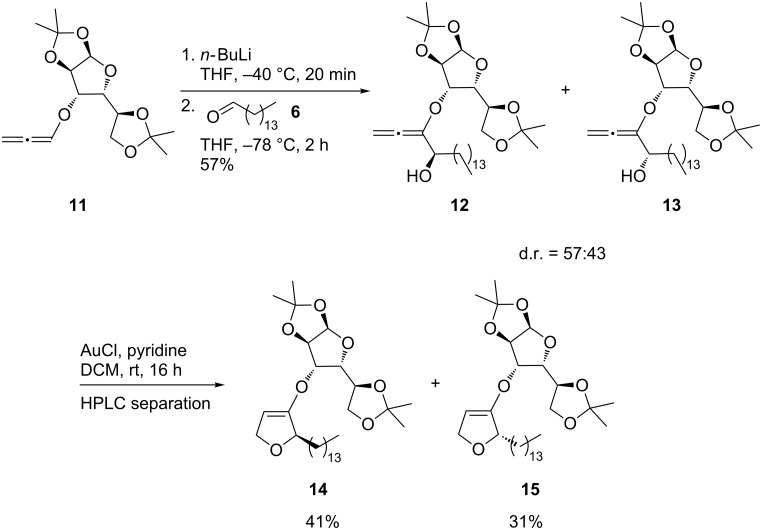
Synthesis of dihydrofurans **14** and **15**.

Having established suitable conditions for the construction of the functionalized tetrahydrofuran core we focused our attention on the synthesis of enantiopure jaspine B (**1**). Earlier investigations by Goré [38] and our group already showed the feasibility of alkoxyallenes with carbohydrate-derived chiral auxiliaries for the synthesis of enantiopure natural products such as preussin [39] and anisomycin [40] or in hetero Diels–Alder reactions [41–42]. For the stereodivergent synthesis of both enantiomers of jaspine B (**1**), we chose diacetoneglucose-derived alkoxyallene **11**, which is easily accessible on gram scale in two steps, starting from propargyl bromide (Scheme 5) [43]. Lithiation of **11** with *n-*BuLi and subsequent addition to pentadecanal (**6**) in analogous fashion to the synthesis of allenyl alcohol **7** led after column chromatography to a 57:43 mixture of the diastereomeric allenyl alcohols **12** and **13** in 57% yield. Base catalysis at higher temperatures, which was successfully used for the cyclization of **7**, apparently induced partial decomposition of the auxiliary and led to only moderate yields (46%) of dihydrofurans **14** and **15**. Better yields of **14** and **15** were achieved under mild reaction conditions employing gold(I) catalysis [44], which after purification and separation afforded the expected products in 41% and 31% yields, respectively. The dihydrofurans **14** and **15** are more stable than the allenyl alcohols **12** and **13** and therefore allowed smooth separation by HPLC. At this point, the assignment of the absolute configuration at C-2 of **14** and **15** was only based on the comparison of reactions earlier studied in our group [45]. They could later be confirmed by comparison of the optical rotation of the final products with values reported in the literature.

**Scheme 6:**
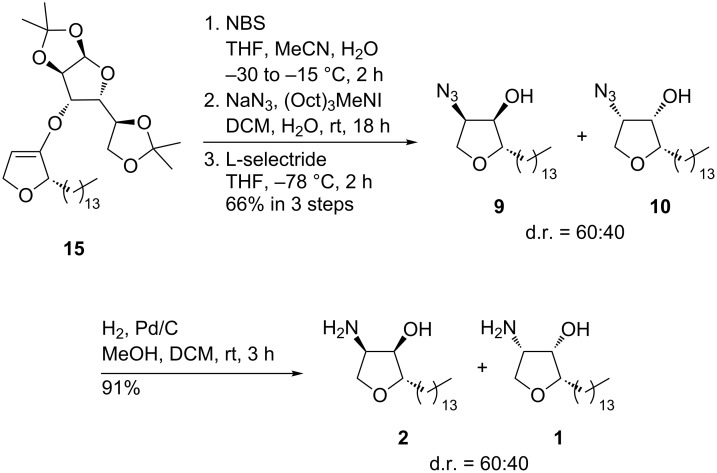
Synthesis of jaspine B (**1**) and its (2*S,*3*R,*4*R*)-diastereomer **2**.

Unfortunately, the attempted functionalization of dihydrofurans **14** and **15** by oxidative azidation (see above) only led to very low yields of the desired α-azidotetrahydrofuranones (6%). The conditions obviously cause decomposition of the auxiliary. As an alternative, the electrophilic bromination of **15** with NBS and the subsequent substitution with sodium azide furnished the desired diastereomeric α-azidotetrahydrofuranones in a ratio comparable to that of the direct oxidative azidation of **8** (Scheme 6). Slightly higher selectivities in favor of the *trans*-configured α-azidofuranone could be observed when the substitution reaction was run for a shorter period. This indicates that a (partial) epimerization at C-4 occurs under the reaction conditions employed. The diastereoselective reduction of the carbonyl group with L-selectride afforded the α-azidotetrahydrofuranones **9** and **10** in 66% yield over three steps. Subsequent hydrogenolysis of **9** and **10** afforded a mixture of jaspine B (**1**) and its (2*S,*3*R,*4*R*)-diastereomer **2** in 91% yield.

**Scheme 7:**
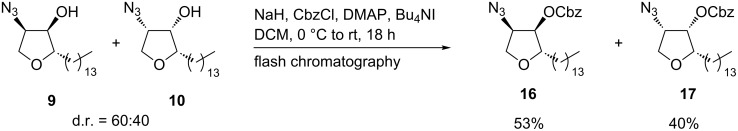
Protection and separation of the diastereomers.

Unfortunately, none of the diastereomeric mixtures obtained in all steps depicted in Scheme 6 were easily separable by HPLC, either due to the instability of the obtained compounds or the similar *R*_f_ values of the tetrahydrofuranol pairs **9/10** and **1/2**. In order to solve this problem and to obtain pure stereoisomers, the α-azidotetrahydrofuranols **9**/**10** were treated with benzyl chloroformate and converted into carbonates **16** and **17**. This protection allowed the smooth separation of the diastereomers by simple flash chromatography, probably due to an enhanced difference of dipole moments and hence *R*_f_ values of the two isomers (Scheme 7).

**Scheme 8:**
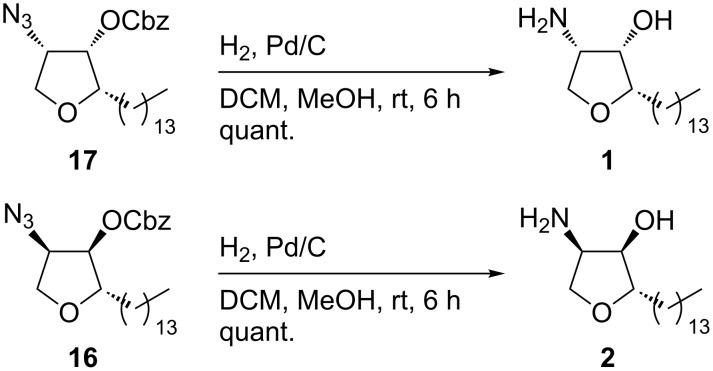
Reduction of the separated diastereomers leading to jaspine B (**1**) and its diastereomer **2**.

Final hydrogenolysis of the separated diastereomers **16** and **17** led to deprotection of the hydroxy group and simultaneous reduction of the azido group. Jaspine B (**1**) and its (2*S,*3*R,*4*R*)-diastereomer **2** were each obtained in quantitative yield showing the expected optical rotations (Scheme 8).

**Scheme 9:**
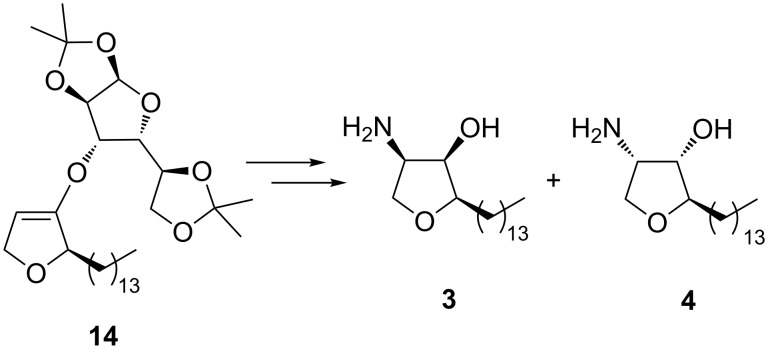
Route to *ent*-jaspine B (**3**) and the (2*R,*3*S,*4*S*)-diastereomer **4**.

Analogously to the reaction sequence leading to **1** and **2**, the synthesis of *ent*-jaspine B (**3**) and the (2*R,*3*S,*4*S*)-diastereomer **4** was also achieved in similar yields employing (2*R*)-configured dihydrofuran **14** as starting material (Scheme 9).

## Conclusion

We developed a stereodivergent synthesis of enantiopure jaspine B and three of its stereoisomers. Important knowledge could also be gained during the preparation of the racemic compounds. The carbohydrate-derived alkoxyallene **11** proved to be useful as a chiral C_3_-building block and allowed the synthesis of both enantiomers of the natural product in a fairly short manner (seven steps starting from the allene). The 5*-endo*-cyclization of allenyl alcohols **12** and **13** by gold catalysis afforded a separable mixture of diastereomeric dihydrofurans **14** and **15** in good yield. Subsequent functionalization using oxidative azidation, as shown for methoxy-substituted dihydrofuran **8**, did not give the expected α-azidotetrahydrofuranones in acceptable yields, but electrophilic bromination and subsequent substitution with sodium azide turned out to be a good alternative for the synthesis of the desired intermediates. Difficulties of the separation of the diastereomeric tetrahydrofuranols were successfully overcome by protection of the C-3 hydroxy group as carbonate, which enabled an easy chromatographic separation of the diastereomers and a global deprotection/reduction leading to pure stereoisomers **1** and **2**. Although the crucial steps of our approach to jaspine B are not stereoselective we found a simple access to the natural product and three of its stereoisomers, demonstrating again the usefulness of intermediates obtained from alkoxyallenes as C_3_-building block [46–50]. Employing other aldehydes instead of pentadecanal our straightforward route should also allow the synthesis of analogues of jaspine B.

## Supporting Information

Experimental details and NMR spectra of all new products and the final compounds are available in the Supporting information.

File 1 Experimental procedures and analytical data and NMR spectra.
