# Supplementary material for: Stereodivergent synthesis of jaspine B and its isomers using a carbohydrate-derived alkoxyallene as C3-building block
Source: Beilstein J Org Chem. 2013 Nov 19;9:2564–9. doi: 10.3762/bjoc.9.291 (PMC3869275; doi:10.3762/bjoc.9.291)

Supporting Information

for

Stereodivergent synthesis of jaspine B and its isomers using a carbohydrate-derived alkoxyallene as C_3-building block

Volker M. Schmiedel, Stefano Stefani and Hans-Ulrich Reissig*

Address: [1]Institut für Chemie und Biochemie, Freie Universität Berlin, Takustr. 3, D-14195 Berlin, Germany

Email: Hans-Ulrich Reissig* - hreissig@chemie.fu-berlin.de

* Corresponding author

Experimental procedures and analytical data and NMR spectra.

Table of contents:

- General information P2
- Experimental procedures P3
- References P14
- Copies of NMR spectra P15

General information

Reactions were generally performed under an argon atmosphere in flame-dried flasks. Solvents and reagents were added by syringe. Solvents were dried with a MB SPS-800-dry solvent system. The reagents were purchased and used as received without further purification unless stated otherwise. Products were purified by flash chromatography on silica gel (230–400 mesh, Merck or Macherey & Nagel). Unless stated otherwise, yields refer to analytically pure samples. NMR spectra were recorded with Bruker (AC 250, AC 500, AVIII 700) and JOEL (ECX 400, Eclipse 500) instruments. Chemical shifts are reported relative to TMS or solvent residual peaks (^{1}H NMR: δ = 0.00 ppm [TMS], δ = 3.31 ppm [CD_3OD], δ = 7.26 ppm [$CDCl_3$]; ^{13}C NMR: δ = 49.0 ppm [CD_3OD], δ = 77.0 ppm [$CDCl_3$]). Integrals are in accordance with assignments and coupling constants are given in Hertz. All ^{13}C NMR spectra are proton-decoupled. For detailed peak assignments 2D spectra were measured (COSY, HMQC, HMBC). IR spectra were measured with a Nexus FT-IR spectrometer fitted with a Nicolet Smart DuraSample IR ATR. MS and HRMS analyses were performed with an Agilent 6210 (ESI-TOF, 4 kV) instrument. Elemental analyses were obtained with a Vario EL or a Vario EL III (Elementar Analysensysteme GmbH) instrument. Melting points were measured with a Reichert apparatus (Thermovar) and are uncorrected. Optical rotations ($[\alpha]_D$) were determined with a Perkin–Elmer 241 polarimeter at the temperatures given.

Typical procedure for the preparation of α-hydroxyallenes by addition of lithiated alkoxyallenes to aldehydes (Procedure 1):

A solution of the corresponding allene (3 equiv) in dry THF (0.7 mL/mmol) at −40 °C was treated with *n*-BuLi (2.5 M in hexane, 2.8 equiv). After 20 min the reaction mixture was cooled to −78 °C and a solution of the aldehyde (1 equiv) in dry THF (3 mL/mmol) was added dropwise. After 3 h of stirring at −78 °C the reaction was quenched with sat. aq. $NaHCO_3$ solution (3 mL/mmol). After warm-up to rt the layers were separated, followed by extraction with ethyl acetate (2 x 3 mL/mmol). The combined organic layers were dried with Na_2SO_4, filtered and the solvent was removed under reduced pressure.

Typical procedure for the hydrogenolysis of carbonates and azides with hydrogen and palladium on charcoal (Procedure 2):

A with hydrogen gas saturated suspension of palladium on charcoal (10%, 50 wt %) in MeOH (25 mL/mmol) was treated with a solution of the corresponding carbonate or azide in dichloromethane (12.5 mL/mmol). The reaction mixture was stirred under hydrogen atomsphere for 6 h at rt and then filtered through a short pad of Celite. The pad was washed several times with dichloromethane and the filtrate was concentrated under reduced pressure to give the corresponding amino alcohol.

3-Methoxyoctadeca-1,2-dien-4-ol (7):

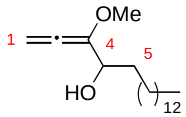

According to procedure 1, methoxyallene (**5**) (4.31 g, 61.5 mmol), *n*-BuLi (23 mL, 57.5 mmol, 2.5 M in hexane), pentadecanal (**6**) (4.64 g, 20.5 mmol) and THF (100 mL) were treated as described. After removal of the solvent, α-hydroxy

allene **7** (6.06 g, ~90% pure) was obtained as a red oil. Due to its instability, the crude product was used without further purification.

^{1}H NMR (400 MHz, $CDCl_3$) δ 0.87 (t, *J* = 6.7 Hz, 3H, CH_3), 1.24 (s_{br}, 24H, CH_2), 1.49–1.67 (m, 2H, 5-H), 3.44 (s, 3H, OCH_3), 4.14 (ddd, *J* = 7.3, 5.8, 4.0 Hz, 1H, 4-H), 5.44–5.59 (m, 2H, 1-H) ppm, the OH signal could not be detected; ^{13}C NMR (101 MHz, $CDCl_3$) δ 14.4 (q, CH_3), 22.8, 25.6, 29.5, 29.6, 29.70, 29.73, 29.78*, 29.81, 29.82*, 32.1, 34.6 (11 t, CH_2), 56.7 (q, OCH_3), 63.2 (d, C-4), 92.3 (t, C-1), 136.2 (s, C-3), 197.1 (s, C-2) ppm, * higher intensity. No further analysis was carried out.

3-Methoxy-2-tetradecyl-2,5-dihydrofuran (8):

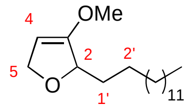

To a solution of α-hydroxyallene **7** (6.06 g, ~90% pure, ~18.2 mmol) in DMSO (100 mL) was added at room temperature potassium *tert*-butoxide (1.15 g, 10.2 mmol). The mixture was heated to 60 °C for 1 h and then water (100 mL) and ethyl acetate (100 mL) were added. The layers were separated at room temperature, followed by extraction of the aqueous layer with ethyl acetate (2 x 70 mL). The combined organic layers were dried with Na_2SO_4, filtered and the solvent was removed under reduced pressure. After purification by column chromatography (silica gel, hexanes/ethyl acetate 30:1), dihydrofuran **8** (4.52 g, 74% in 2 steps) was obtained as a yellowish oil.

^{1}H NMR (400 MHz, C_6D_6) δ 0.85 (t, *J* = 6.9 Hz, 3H, CH_3), 1.24 (s_{br}, 22H, CH_2), 1.47–1.78 (m, 3H, 2'-H, 1'-H), 1.79–1.90 (m, 1H, 1'-H), 3.12 (s, 3H, OCH_3), 4.15 (q, *J* = 1.6 Hz, 1H, 2-H), 4.53–4.62 (m, 2H, 5-H), 4.74 (m_c, 1H, 4-H) ppm; ^{13}C NMR (101 MHz, C_6D_6) δ 14.3 (q, CH_3), 23.2, 25.2, 29.9*, 30.15, 30.16, 30.18*, 30.19, 30.21, 30.25, 32.4 (10 t, CH_2), 34.6 (t, C-1'), 56.9 (q, OCH_3), 72.9 (t, C-5), 81.8 (d, C-2), 90.5 (d, C-4), 159.1 (s, C-3) ppm, * higher intensity; IR (KBr) ν:

2990–2860 (C-H), 1650 (C=C), 1110–1090 (C-O) cm^{-1}; ESI-TOF (*m/z*): $[M + Na]^+$ calcd for $C_{19}H_{36}NaO_2$, 319.2613. found: 319.2601.

rac-4-Azido-2-(tetradecyl)tetrahydrofuran-3-ols (_rac_-9/_rac_-10):

A solution of dihydrofuran **8** (44 mg, 0.15 mmol) in acetonitrile (0.5 mL) at −25 °C was treated with sodium azide (43 mg, 0.67 mmol). After stirring for 5 min a solution of ceric(IV) ammonium nitrate (243 mg, 0.44 mmol) in acetonitrile (1.5 mL) was added and the mixture was stirred at −25 °C for 2.5 h. Diethyl ether (2 mL) and water (2 mL) were added and after warm-up to rt the phases were separated. The organic phase was washed with water (2 x 2 mL), the combined organic layers were dried with Na_2SO_4, filtered and the solvents were removed under reduced pressure, to give the crude azido furanones which were used without further purification.

The crude product was dissolved in THF (2.5 mL) and at −78 °C treated dropwise with L-selectride (1 M in THF, 0.2 mL, 0.2 mmol). The mixture was stirred for 2 h, and then quenched with sat. aq. NH_4Cl solution (3 mL). The phases were separated, followed by extraction of the aqueous layer with ethyl acetate (2 x 3 mL). The combined organic layers were dried with Na_2SO_4, filtered and the solvents were removed under reduced pressure. After purification by column chromatography (silica gel, hexanes/ethyl acetate 10:1) a 60:40 mixture of the *(trans,cis)*-tetrahydrofuranol ***rac*-9** and the *(cis,cis)*-tetrahydrofuranol ***rac*-10** was obtained as a colorless solid (29 mg, 62% over two steps).

The spectroscopic data agree with the data of the enantiopure compounds **9** and **10** (see below).

4-Amino-2-(tetradecyl)tetrahydrofuran-3-ols (_rac_-1/_rac_-2):

According to procedure 2, a mixture of *rac*-**9**/*rac*-**10** (40 mg, 0.12 mmol, dr = 60:40), palladium on charcoal (10% Pd, 20 mg), dichloromethane (1 mL) and methanol (2 mL) were treated as described. After purification by column

chromatography (silica gel, dichloromethane/methanol 10:1), an inseparable mixture of *rac*-jaspine B *rac*-**1** and its *(trans,cis)*-diastereomer *rac*-**2** was obtained as a colorless solid (36 mg, 91%, dr = 40:60) (melting area: 85– 90 °C, lit. value for **1** ranges from 83–97 °C,[1] lit. value 93–95 °C).[2]

The spectroscopic data agree with the data of the enantiopure compounds **1** and **2** (see below).

3-{[(3a*R*,5*R*,6*S*,6a*R*)-5-{(*S*)-2,2-Dimethyl-1,3-dioxolan-4-yl}-2,2-dimethyltetrahydro-furo[2,3-*d*][1,3]dioxol-6-yl]oxy}octadeca-1,2-dien-4-ol (12/13):

According to procedure 1, diacetone glucose-derived allene **11** (6.83 g, 22.9 mmol), *n*-BuLi (8.45 mL, 21.1 mmol, 2.5 M in hexane), pentadecanal (**6**) (2.01 g, 8.76 mmol) and THF (40 mL) were treated as described. After purification by column chromatography (silica gel, hexanes/ethyl acetate 7:1, gradient to 5:1) a 57:43 mixture of the (4*R*)- and (4*S*)-diastereomers was obtained as a yellow oil (2.64 g, 57%) (fractions with enriched diastereomeric ratios allowed assignments to the two diastereomers).

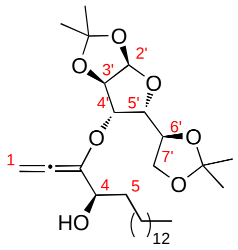

(4*R*)-Diastereomer 12: ^{1}H NMR (500 MHz, $CDCl_3$) δ 0.86 (t, *J* = 7.0 Hz, 3H, CH_3), 1.24–1.30 (m, 24H, CH_2), 1.29, 1.33, 1.41, 1.48 (4 s, 3H each, CH_3), 1.56–1.65 (m, 2H, 5-H), 2.36 (m_c, 1H, OH), 4.01 (dd, *J* = 8.7, 5.1 Hz, 1H, 7'-H), 4.11–4.13 (m, 2H, 4-H, 7'-H), 4.16 (dd, *J* = 8.0, 3.2 Hz, 1H, 5'-H), 4.26 (d, *J* = 3.2 Hz, 1H, 4'-H), 4.33 (ddd, *J* = 8.0, 6.2, 5.1 Hz, 1H, 6'-H), 4.56 (d, *J* = 3.8 Hz, 1H, 3'-H), 5.58–5.59 (m, 2H, 1-H), 5.86 (d, *J* = 3.8 Hz, 1H, 2'-H) ppm; ^{13}C NMR (126 MHz, $CDCl_3$) δ 14.0 (q, CH_3), 22.6, 25.1 (2 t, CH_2), 25.3, 26.1, 26.65, 26.68 (4 q, CH_3),

29.2, 29.3*, 29.46, 29.48, 29.52, 29.53, 29.56, 29.57, 31.8 (9 t, CH_2), 34.4 (t, C-5), 67.3 (t, C-7'), 71.1 (d, C-4), 72.5 (d, C-6'), 80.6 (d, C-5'), 81.0 (d, C-4'), 82.3 (d, C-3'), 92.3 (t, C-1), 105.1 (d, C-2'), 109.1, 111.9 (2 s, $C(CH_3)_2$), 133.1 (s, C-3), 196.7 (s, C-2) ppm, * higher intensity.

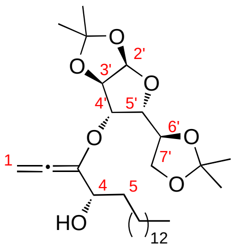

(4*S*)-Diastereomer 13: ^{1}H NMR (500 MHz, $CDCl_3$) δ 0.87 (t, *J* = 7.0 Hz, 3H, CH_3), 1.25–1.30 (m, 24H, CH_2), 1.30, 1.34, 1.41, 1.49 (4s, 3H each, CH_3), 1.54–1.64 (m, 2H, 5-H), 2.44 (dd, *J* = 4.8, 2.4 Hz, 1H, OH), 4.02 (dd, *J* = 8.7, 5.1 Hz, 1H, 7'-H), 4.10–4.13 (m, 2H, 4-H, 7'-H), 4.17 (dd, *J* = 8.1, 3.4 Hz, 1H, 5'-H), 4.26 (d, *J* = 3.4 Hz, 1H, 4'-H), 4.31–4.37 (m, 1H, 6'-H), 4.57 (d, *J* = 3.8 Hz, 1H, 3'-H), 5.56–5.66 (m, 2H, 1-H), 5.87 (d, *J* = 3.8 Hz, 1H, 2'-H) ppm; ^{13}C NMR (126 MHz, $CDCl_3$) δ 14.3 (q, CH_3), 22.8 (t, CH_2), 25.4 (q, CH_3), 25.7 (t, CH_2), 26.4, 26.94, 26.96 (3 q, CH_3), 29.5, 29.66, 29.75, 29.76, 29.80, 29.81, 29.84*, 32.1, 34.2 (9 t, CH_2), 67.6 (t, C-7'), 71.2 (d, C-4), 72.8 (d, C-6'), 80.9 (d, C-5'), 81.3 (d, C-4'), 82.6 (d, C-3'), 93.0 (t, C-1), 105.5 (d, C-2'), 109.4, 112.1 (2 s, $C(CH_3)_2$), 133.9 (s, C-3), 196.9 (s, C-2) ppm, * higher intensity.

Data of mixture of diastereomers: IR (ATR) ν: 3455 (O-H), 2925–2855 (C-H), 1730 (C=C) cm^{-1}; ESI-TOF (*m/z*): $[M + Na]^+$ calcd for $C_{30}H_{52}NaO_7$ 547.3611, found 547.3578.

(3a*R*,5*R*,6*S*,6a*R*)-5-[(*S*)-2,2-Dimethyl-1,3-dioxolan-4-yl]-2,2-dimethyl-6-[(2-tetra-decyl-2,5-dihydrofuran-3-yl)oxy]tetrahydrofuro[2,3-*d*][1,3]dioxole (14/15):

A solution of α-hydroxy allenes **12/13** (1.17 g, 2.23 mmol, ≈6:4 mixture of diastereomers, determined by ^{1}H NMR spectroscopy) and pyridine (0.03 mL,

0.33 mmol) in dichloromethane (34 mL) was treated at rt with gold(I) chloride (26 mg, 0.11 mmol) and stirred for 14 h. Gold(I) chloride (13 mg, 0.055 mmol) was added again and the mixture was stirred for another 2 h. A 1:1 mixture of sat. aq. $NaHSO_4$ solution and water (34 mL) was added. The layers were separated, followed by extraction of the aqueous layer with dichloromethane (2 x 25 mL). The combined organic layers were dried with Na_2SO_4, filtered and the solvent was removed under reduced pressure. After purification by column chromatography (silica gel, hexanes/ethyl acetate 5:1) the obtained mixture of two diastereomers was separated by HPLC (7% ethyl acetate in hexanes, 96 mL/min, Nu 50-5 column). The (2*R*)-diastereomer **14** (475 mg, 41%) and (2*S*)-diastereomer **15** (360 mg, 31%) were obtained as yellowish oils.

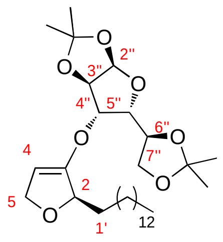

(2*R*)-Diastereomer 14: $[\alpha]_D^{23}$ -26.8 (*c* 1.0, $CHCl_3$); ^1H NMR (500 MHz, $CDCl_3$) δ 0.86 (t, *J* = 6.9 Hz, 3H, CH_3), 1.23 (s_{br}, 22H, CH_2), 1.31 (s, 6H, 2 CH_3), 1.32–1.39 (m, 2H, 2'-H), 1.41 (s, 3H, CH_3), 1.45–1.56 (m, 4H, CH_3, 1'-H), 1.61–1.69 (m, 1H, 1'-H), 3.98 (dd, *J* = 8.5, 5.8 Hz, 1H, 7''-H), 4.05–4.11 (m, 1H, 7''-H), 4.18 (dd, *J* = 7.9, 3.2 Hz, 1H, 5''-H), 4.27 (dt, *J* = 7.9, 5.8 Hz, 1H, 6''-H), 4.38 (d, *J* = 3.2 Hz, 1H, 4''-H), 4.53–4.58 (m, 1H, 2-H), 4.61 (d, *J* = 3.9 Hz, 1H, 3''-H), 4.62 (d, *J* = 1.6 Hz, 2H, 5-H), 4.79 (t, *J* = 1.6 Hz, 1H, 4-H), 5.87 (d, *J* = 3.9 Hz, 1H, 2''-H) ppm; ^{13}C NMR (126 MHz, $CDCl_3$) δ 14.2 (q, CH_3), 22.8, 24.3 (2 t, CH_2), 25.2, 26.3, 26.7, 26.8 (4 q, CH_3), 29.4, 29.72, 29.74, 29.76*, 29.82, 29.9, 32.0 (7 t, CH_2), 33.9 (t, C-1'), 67.4 (t, C-7''), 72.1 (d, C-6''), 72.9 (d, C-3''), 80.5 (d, C-5''), 81.5 (d, C-2), 81.8 (t, C-5), 82.0 (d, C-4''), 92.2 (d, C-4), 105.3 (d, C-2''), 109.3, 112.1 (2 s, $C(CH_3)_2$), 154.8 (s, C-3) ppm, * higher intensity; IR (ATR) ν: 2980–2850 (C-H), 1660 (C=C) cm^{-1}; ESI-TOF *(m/z)*: $[M + Na]^+$ calcd for $C_{30}H_{52}NaO_7$ 547.3611; found 547.3611; Anal. calcd for $C_{30}H_{52}O_7$ (524.7): C, 68.67; H, 9.99; found: C, 69.07; H, 9.95.

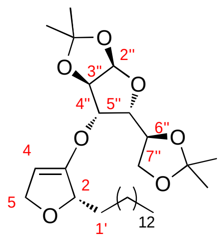

(2*S*)-Diastereomer 15: $[\alpha]_D^{23}$ -22.4 (*c* 1.0, $CHCl_3$); ^{1}H NMR (500 MHz, $CDCl_3$) δ 0.87 (t, *J* = 6.9 Hz, 3H, CH_3), 1.24 (s_{br}, 22H, CH_2), 1.32, 1.33 (2 s, 3H each, CH_3), 1.34–1.37 (m, 2H, 2'-H), 1.42 (s, 3H, CH_3), 1.44–1.49 (m, 1H, 1'-H), 1.51 (s, 3H, CH_3), 1.56–1.66 (m, 1H, 1'-H), 4.00–4.10 (m, 2H, 7''-H), 4.23 (dd, *J* = 7.4, 3.1 Hz, 1H, 5''-H), 4.30 (dt, *J* = 7.4, 5.6 Hz, 1H, 6''-H), 4.40 (d, *J* = 3.1 Hz, 1H, 4''-H), 4.58–4.62 (m, 3H, 3''-H, 5-H), 4.63–4.67 (m, 1H, 2-H), 4.79 (d, *J* = 1.5 Hz, 1H, 4-H), 5.85 (d, *J* = 3.8 Hz, 1H, 2''-H) ppm; ^{13}C NMR (126 MHz, $CDCl_3$) δ 14.2 (q, CH_3), 22.8, 24.6 (2 t, CH_2), 25.4, 26.4, 26.8, 26.9 (4 q, CH_3), 29.4, 29.69, 29.73*, 29.75*, 29.77*, 32.0, 33.9 (7 t, CH_2), 66.9 (t, C-7''), 72.3 (d, C-6''), 72.9 (d, C-3''), 80.1 (d, C-5''), 81.3 (d, C-2), 81.8 (t, C-5), 81.8 (d, C-4''), 92.5 (d, C-4), 105.2 (d, C-2''), 109.3, 112.2 (2 s, *C*$(CH_3)_2$), 154.9 (s, C-3) ppm, * higher intensity; IR (ATR) ν: 2985–2850 (C-H), 1660 (C=C) cm^{-1}; ESI-TOF *(m/z):* $[M + Na]^+$ calcd for $C_{30}H_{52}NaO_7$ 547.3611; found 547.3609; Anal. calcd for $C_{30}H_{52}O_7$ (524.7): C, 68.67; H, 9.99; found C, 68.71; H, 9.98.

(2*S*)-4-Azido-2-(tetradecyl)tetrahydrofuran-3-ols 9/10:

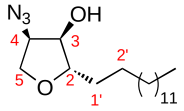

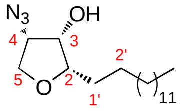

N-Bromosuccinimide (163 mg, 0.914 mmol) was dissolved in a mixture of acetonitrile (4.5 mL), THF (2 mL) and water (0.45 mL) at −30 °C. After 5 min a solution of dihydrofuran **15** (400 mg, 0.762 mmol) in THF (2.5 mL) was added dropwise. Over a period of 2 h the reaction mixture was allowed to warm to −15 °C and then quenched with water (4 mL). The layers were separated and the aqueous layer was extracted with diethylether (3 x 4 mL). The combined organic layers were dried with Na_2SO_4, filtered and the solvents were removed under

reduced pressure to give a crude bromo-substituted furanone. This was used without further purification and dissolved in dichloromethane (2 mL) at rt. Solutions of trioctylmethylammonium chloride (13 mg, 0.035 mmol) and sodium azide (239 mg, 3.67 mmol) in water (1 mL) were added. After stirring for 18 h, the mixture was quenched with water (1 mL). The layers were separated and the aqueous layer was extracted with dichloromethane (1 x 2 mL). The combined organic layers were dried with Na_2SO_4, filtered and the solvent was removed under reduced pressure to give a crude azido furanone. This was used without further purification, dissolved in dry THF (7.5 mL) and at −78 °C L-selectride (1.5 mL, 1.5 mmol, 1 M in THF) was added. After stirring for 2 h at −78 °C, the reaction mixture was quenched with sat. aq. NH_4Cl solution (7.5 mL). The layers were separated, followed by extraction of the aqueous layer with ethyl acetate (3 x 5 mL). The combined organic layers were dried with Na_2SO_4, filtered and the solvents were removed under reduced pressure. The obtained crude product was purified by column chromatography (silica gel, hexanes/ethyl acetate 10:1). An inseparable 57:43 mixture of the (2*S*,3*R*,4*R*):(2*S*,3*S*,4*S*)-diastereomers of tetrahydrofuranols **9** and **10** (163 mg, 66% over 3 steps) was obtained as a colorless solid.

(2*S*,3*R*,4*R*)-Diastereomer 9: ^{1}H NMR (400 MHz, $CDCl_3$) δ 0.87 (t, *J* = 6.9 Hz, 3H, CH_3), 1.18–1.45 (m, 24H, CH_2), 1.60–1.65 (m, 2H, 1'-H), 2.03 (s_{br}, 1H, OH), 3.58–3.66 (m, 1H, 2-H), 3.81 (dd, *J* = 9.6, 4.5 Hz, 1H, 5-H), 3.87–3.93 (m, 1H, 3-H), 4.05 (td, *J* = 5.7, 4.5 Hz, 1H, 4-H), 4.12 (dd, *J* = 9.6, 5.7 Hz, 1H, 5-H) ppm.

(2*S*,3*S*,4*S*)-Diastereomer 10: ^{1}H NMR (400 MHz, $CDCl_3$) δ 0.87 (t, *J* = 6.9 Hz, 3H, CH_3), 1.18–1.45 (m, 24H, CH_2), 1.60–1.65 (m, 2H, 1'-H), 2.07 (s_{br}, 1H, OH), 3.76 (td, *J* = 6.9, 3.7 Hz, 1H, 2-H), 3.85 (dd, *J* = 9.2, 7.0 Hz, 1H, 5-H), 3.98 (dd, *J* = 9.2, 7.0 Hz, 1H, 5-H), 4.11 (td, *J* = 7.0, 4.9 Hz, 1H, 4-H), 4.20 (dd, *J* = 4.9, 3.7 Hz, 1H, 3-H) ppm.

The spectroscopic data previously published[3] allowed assignment of the signals to the two diastereomers.

(2*S*)-4-Azido-2-(tetradecyl)tetrahydrofuran-3-yl benzyl carbonates (16/17): A solution of furanols **9/10** (40 mg, 0.123 mmol, dr = 57:43) in dichloromethane (0.5 mL) was treated dropwise at 0 °C with a suspension of sodium hydride (27 mg, 0.676 mmol, 60% in mineral oil) in dichloromethane (0.5 mL). DMAP (8 mg, 0.061 mmol) and tetra-*n*-butylammonium iodide (45 mg, 0.123 mmol) were added and the reaction mixture was stirred for 5 min. Benzyl chloroformate (95%, 0.02 mL, 0.123 mmol) was added dropwise and the mixture was allowed to warm to rt over a period of 16 h. The reaction mixture was quenched with sat. aq. NH_4Cl solution (1 mL), followed by extraction with dichloromethane (3 x 1 mL). The combined organic layers were dried with Na_2SO_4, filtered and the solvent was removed under reduced pressure. Separation by column chromatography (silica gel, hexanes/ethyl acetate 20:1) provided pure (2*S*,3*R*,4*R*)-**16** (30 mg, 53%) and pure (2*S*,3*S*,4*S*)-**17** (22 mg, 40%) as colorless oils.

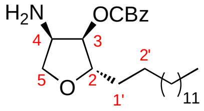

(2*S*,3*R*,4*R*)-Diastereomer (16): $[\alpha]_D^{23}$ +5.1 (*c* 0.52, $CHCl_3$); ^{1}H NMR (500 MHz, $CDCl_3$) δ 0.88 (t, *J* = 6.9 Hz, 3H, CH_3), 1.26 (s_{br}, 22H, CH_2), 1.37–1.46 (m, 1H, 2'-H), 1.50–1.55 (m, 1H, 2'-H), 1.55–1.68 (m, 2H, 1'-H), 3.71–3.75 (m, 1H, 5-H), 3.90 (dt, *J* = 7.9, 5.6 Hz, 1H, 2-H), 4.11–4.18 (m, 2H, 4-H, 5-H), 4.77 (t, *J* = 5.6 Hz, 1H, 3-H), 5.21 (d, *J* = 12.1 Hz, 1H, CH_2Ph), 5.24 (d, *J* = 12.1 Hz, 1H, CH_2Ph), 7.30–7.43 (m, 5H, Ph) ppm; ^{13}C NMR (126 MHz, $CDCl_3$) δ 14.2 (q, CH_3), 22.8, 25.6, 29.46, 29.56, 29.57, 29.65, 29.73, 29.76*, 29.79, 29.80, 32.0 (11 t, CH_2), 33.3 (t, C-1'), 60.7 (d, C-4), 69.7 (t, C-5), 70.4 (t, CH_2Ph), 80.0 (d, C-3), 81.0 (d, C-2), 128.4, 128.7, 128.8 (3 d, Ph), 134.9 (s, Ph), 154.6 (s, C=O) ppm; * higher intensity; ESI-TOF *(m/z)*: $[M + Na]^+$ calcd for $C_{26}H_{41}N_3NaO_7$ 482.2990; found 482.3004.

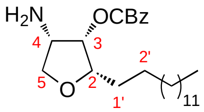

(2*S*,3*S*,4*S*)-Diastereomer (17): $[\alpha]_D^{23}$ -5.3 (*c* 0.4, $CHCl_3$); ^{1}H NMR (500 MHz, $CDCl_3$) δ 0.88 (t, *J* = 6.9 Hz, 3H, CH_3), 1.25 (s_{br}, 22H, CH_2), 1.35–1.44 (m, 1H, 2'-H), 1.49–1.69 (m, 3H, 1'-H, 2'-H), 3.82 (dd, *J* = 9.1, 7.2 Hz, 1H, 5-H), 3.94 (ddd, *J* = 8.2, 5.4, 4.1 Hz, 1H, 2-H), 4.00 (dd, *J* = 9.1, 7.2 Hz, 1H, 5-H), 4.15 (td, *J* = 7.2, 5.1 Hz, 1H, 4-H), 5.19–5.28 (m, 3H, *CH_2*Ph, 3-H), 7.30–7.45 (m, 5H, Ph) ppm; ^{13}C NMR (126 MHz, $CDCl_3$) δ 14.2 (q, CH_3), 22.8, 26.0, 29.1, 29.45, 29.54, 29.59, 29.64, 29.73, 29.75, 29.77, 29.78*, 32.0 (12 t, CH_2), 61.8 (d, C-4), 68.7 (t, C-5), 70.2 (t, *C*H_2Ph), 77.4 (d, C-3), 80.6 (d, C-2), 128.3, 128.5, 128.7 (3 d, Ph), 135.1 (s, Ph), 154.9 (s, C=O) ppm, * higher intensity; ESI-TOF *(m/z)*: $[M + Na]^+$ calcd for $C_{26}H_{41}N_3NaO_7$ 482.2990; found 482.2999.

(2*S*,3*R*,4*R*)-4-Amino-2-(tetradecyl)tetrahydrofuran-3-ol (2):

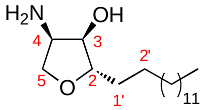

According to procedure 2, carbonate **16** (32.0 mg, 0.069 mmol), palladium on charcoal (10% Pd, 16 mg), MeOH (2 mL) and dichloromethane (1 mL) were treated as described. After removal of the solvents at reduced pressure, (2*S*,3*R*,4*R*)-**2** (20.8 mg, quant.) was obtained as a colorless solid.

mp 91–96 °C, lit. value 93–95 °C;[2] $[\alpha]_D^{29}$ -15.2 (*c* 0.66, MeOH), lit. value -29.6 (*c* 0.48, MeOH);[4] ^{1}H NMR (500 MHz, CD_3OD) δ 0.90 (t, *J* = 6.9 Hz, 3H, CH_3), 1.15–1.41 (m, 24H, CH_2), 1.42–1.68 (m, 4H, 1'-H, 2'-H), 3.61–3.78 (m, 3H, 2-H, 4-H, 5-H), 3.96–4.07 (m, 1H. 3-H), 4.15 (dd, *J* = 8.0, 3.0 Hz, 1H, 5-H). ^{13}C NMR (126 MHz, CD_3OD) δ 13.1 (q, CH_3), 22.4, 25.6, 29.2, 29.33, 29.35, 29.39, 29.44*, 29.46*, 29.47, 31.8, 32.8 (11 t, CH_2), 52.4 (d, C-4), 68.1 (t, C-5), 73.0 (d, C-3), 83.9 (d, C-2) ppm, * higher intensity; ESI-TOF *(m/z)*: $[M + H]^+$ calcd for $C_{18}H_{38}NO_2$ 300.2903; found 300.2896.

The optical rotation value of the (*2R,3S,4S*)-diastereomer **4** was measured as: $[\alpha]_D^{26}$ +18.4 (*c* 0.61, MeOH).

The spectroscopic data agree with previously published results.[5]

Jaspine B (1):

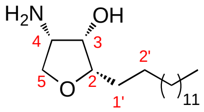

According to procedure 2, carbonate **17** (7.9 mg, 0.017 mmol), palladium on charcoal (10% Pd, 4 mg), MeOH (1 mL) and dichloromethane (0.5 mL) were treated as described. After removal of the solvents under reduced pressure, jaspine B (**1**) (5.2 mg, quant.) was obtained as a colorless solid.

mp 90–92 °C, lit. values range from 83–97 °C;[1] $[\alpha]_D^{27}$ +18.4 (*c* 0.48, MeOH), lit. values +19.7 (*c* 0.5 MeOH)[3], +9.4 (c 0.3, MeOH);[5] ^{1}H NMR (500 MHz, $CDCl_3$) δ 0.87 (t, *J* = 6.9 Hz, 3H, CH_3), 1.18–1.45 (m, 24H, CH_2), 1.61–1.72 (m, 2H, 1'-H), 3.51 (dd, *J* = 8.5, 6.8 Hz, 1H, 5-H), 3.62–3.67 (m, 1H, 4-H), 3.73 (ddd, *J* = 7.6, 6.2, 3.5 Hz, 1H, 2-H), 3.86 (dd, *J* = 5.1, 3.5 Hz, 1H, 3- H), 3.92 (dd, *J* = 8.5, 7.4 Hz, 1H, 5-H) ppm; ^{13}C NMR (126 MHz, $CDCl_3$) δ 14.5 (q, CH_3), 22.9, 26.52, 26.54, 29.62, 29.64, 29.68, 29.72, 29.8*, 29.9*, 30.0, 32.1 (11 t, CH_2), 54.4 (d, C-4), 71.9 (t, C-5), 72.5 (d, C-3), 83.4 (d, C-2) ppm, * higher intensity; ESI-TOF *(m/z)*: $[M + H]^+$ calcd for $C_{18}H_{38}NO_2$ 300.2903; found 300.2899.

The optical rotation value of *ent*-jaspine B (**3**) was measured as: $[\alpha]_D^{26}$ -19.8 (*c* 0.58, MeOH).

The spectroscopic data agree with previously published results.[5]

References

[1a)] K. R. Prasad, A. Chandrakumar, *J. Org. Chem.* **2007**, *72*, 6312–6315, doi: 10.1021/jo0707838.

[1b)] V. Dhand, S. Chang, R. Britton, *J. Org. Chem.* **2013**, doi: org/10.1021/jo4013223.

[2] E. Abraham, E. A. Brock, J. I. Candela-Lena, S. G. Davies, M. Georgiou, R. L. Nicholson, J. H. Perkins, P. M. Roberts, A. J. Russell, E. M. Sanchez-Fernandez, P. M. Scott, A. D. Smith, J. E. Thomson, *Org. Biomol. Chem.* **2008**, *6*, 1665–1673, doi: 10.1039/b801671b.

[3] T. Lee, S. Lee, Y. S. Kwak, D. Kim, S. Kim, *Org. Lett.* **2007**, *9*, 429, doi: 10.1021/ol062756u.

[4] M. Martinková, K. Pomikalová, J. Gonda, M. Vilková, *Tetrahedron* **2013**, *69*, 8228–8244, doi: 10.1016/j.tet.2013.07.028.

[5] E. Abraham, S. G. Davies, P. M. Roberts, A. J. Russell, J. E. Thomson, *Tetrahedron: Asymmetry* **2008**, *19*, 1027–1047, doi: 10.1016/j.tetasy.2008.04.017

^{1}H NMR (400 MHz, C_6D_6) 3-Methoxy-2-tetradecyl-2,5-dihydrofuran (8)

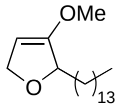

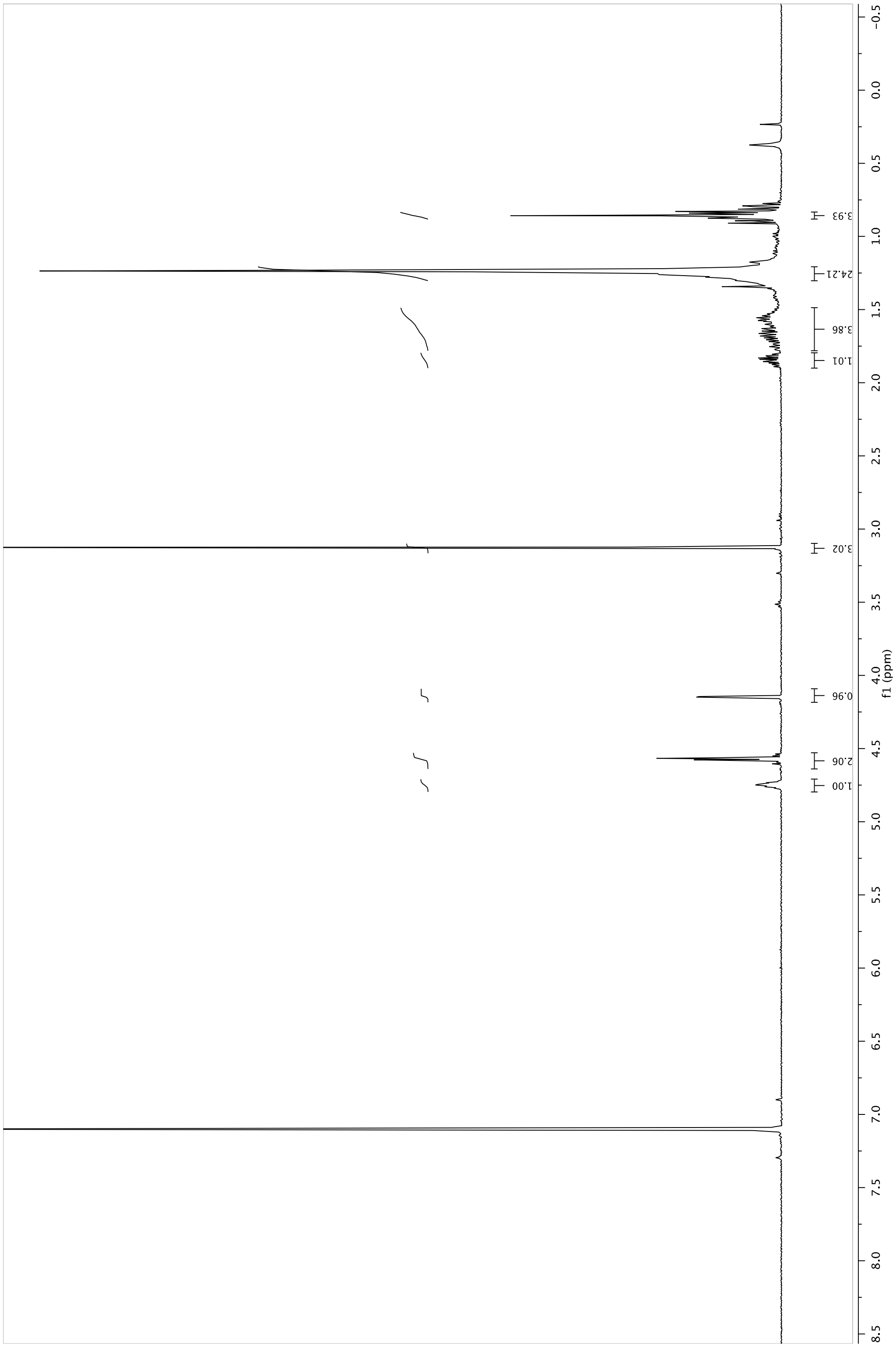

^{13}C NMR (101 MHz, C_6D_6) 3-Methoxy-2-tetradecyl-2,5-dihydrofuran (8)

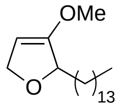

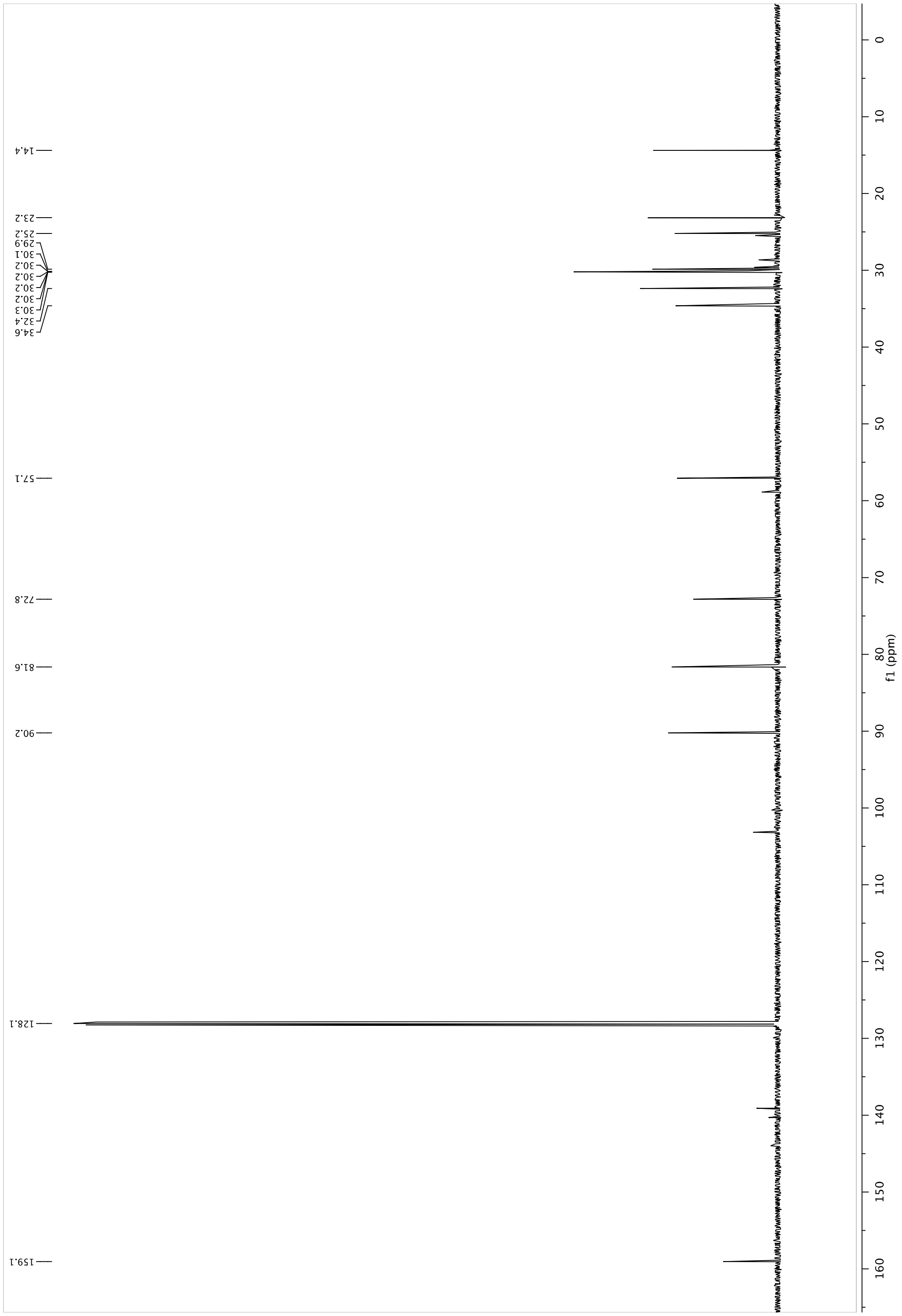

^{1}H NMR (500 MHz, $CDCl_3$) 3-{[(3a*R*,5*R*,6*S*,6a*R*)-5-{(*S*)-2,2-Dimethyl-1,3-dioxolan-4-yl}-2,2-dimethyltetrahydro-furo[2,3-*d*][1,3]dioxol-6-yl]oxy}octadeca-1,2-dien-4-ol (12)

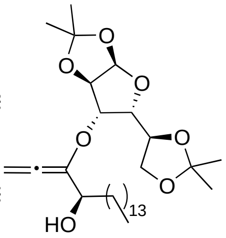

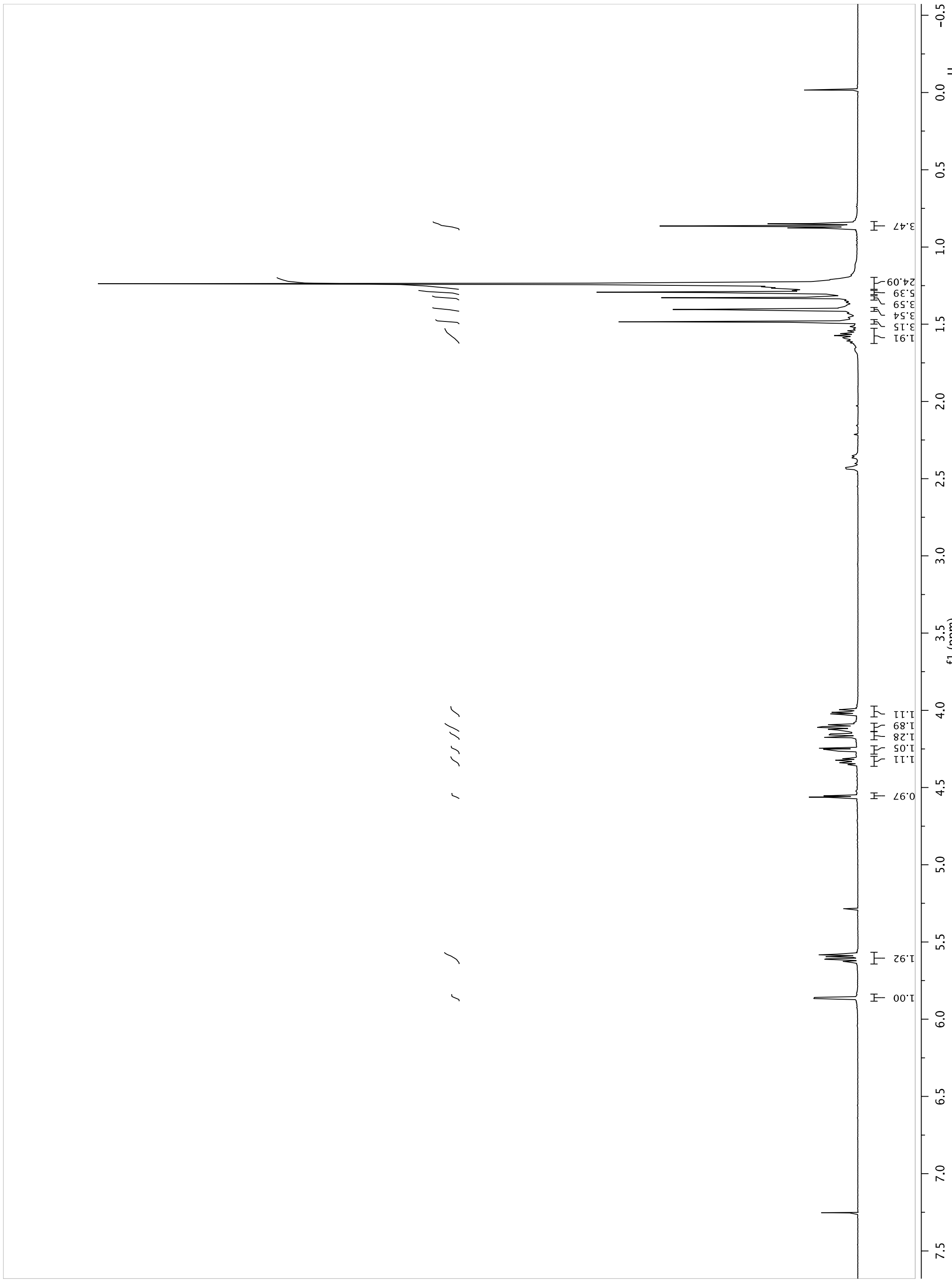

^{13}C NMR (126 MHz, $CDCl_3$) 3-{[(3a*R*,5*R*,6*S*,6a*R*)-5-{(*S*)-2,2-Dimethyl-1,3-dioxolan-4-yl}-2,2-dimethyltetrahydro-furo[2,3-*d*][1,3]dioxol-6-yl]oxy}octadeca-1,2-dien-4-ol (12)

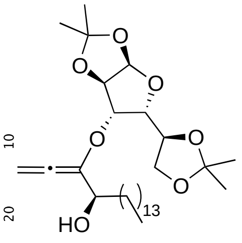

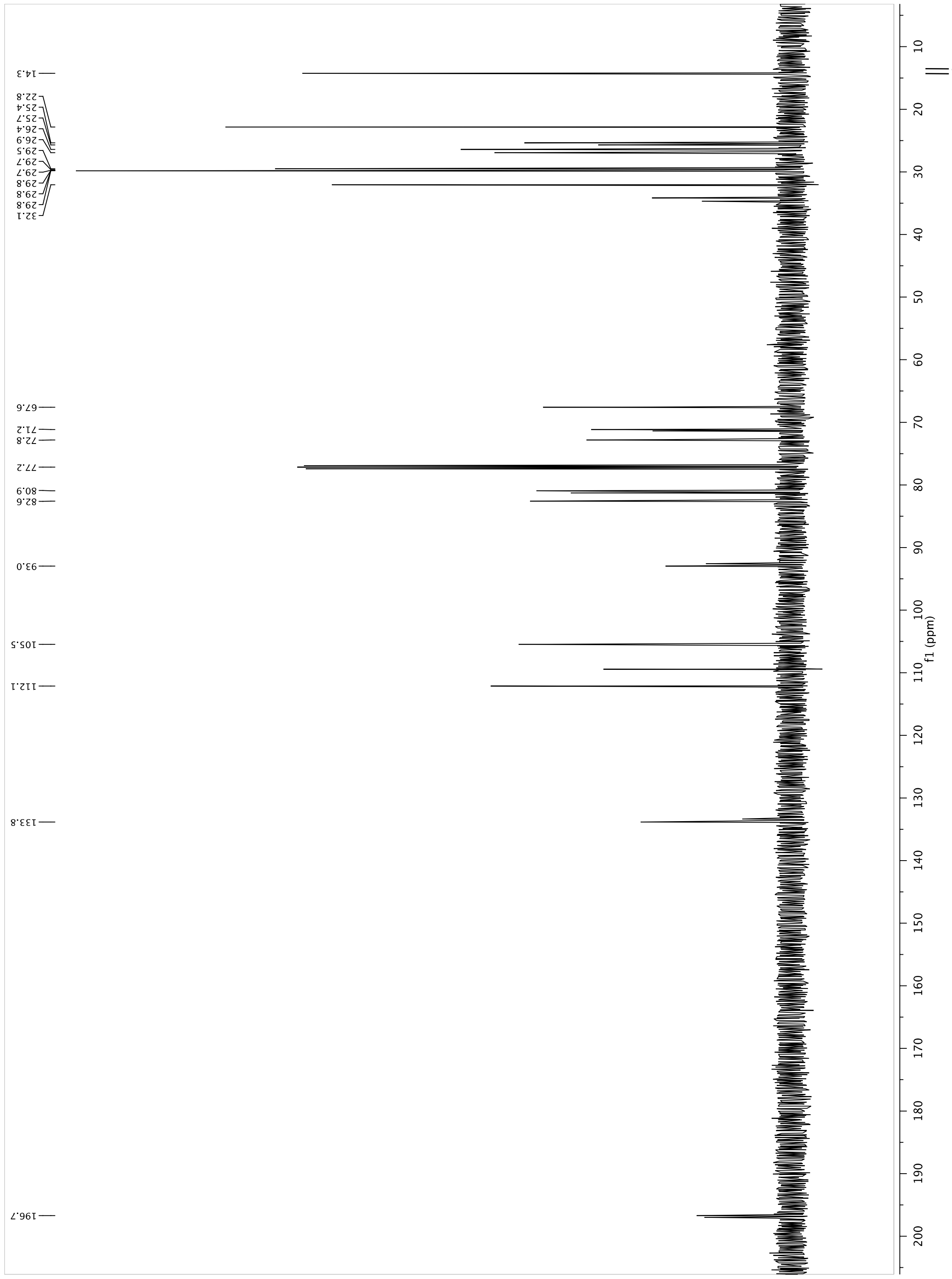

^{1}H NMR (500 MHz, $CDCl_3$) 3-{[(3a*R*,5*R*,6*S*,6a*R*)-5-{(*S*)-2,2-Dimethyl-1,3-dioxolan-4-yl}-2,2-dimethyltetrahydro-furo[2,3-*d*][1,3]dioxol-6-yl]oxy}octadeca-1,2-dien-4-ol (13)

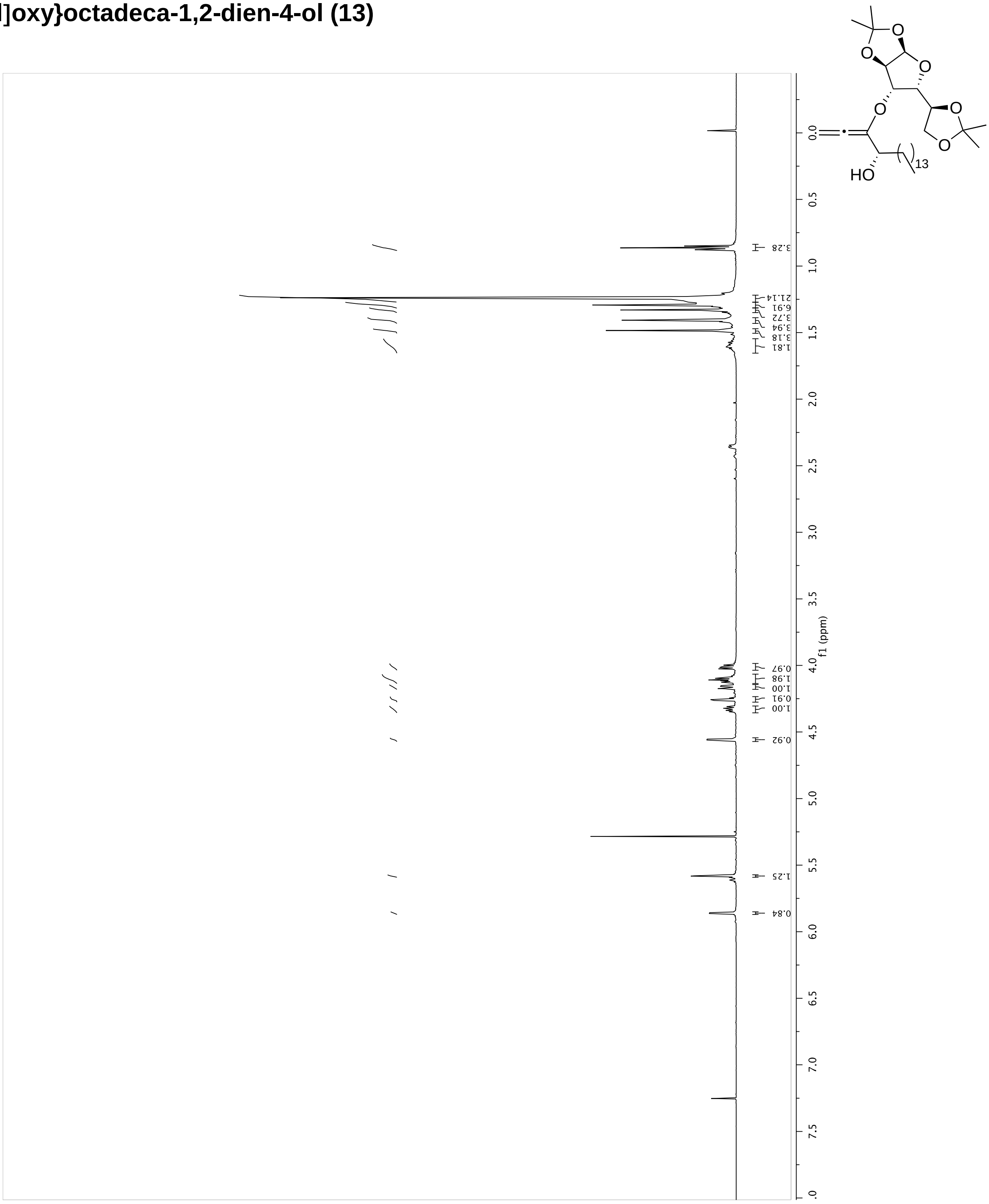

^{13}C NMR (126 MHz, $CDCl_3$) 3-{[(3a*R*,5*R*,6*S*,6a*R*)-5-{(*S*)-2,2-Dimethyl-1,3-dioxolan-4-yl}-2,2-dimethyltetrahydro-furo[2,3-*d*][1,3]dioxol-6-yl]oxy}octa-deca-1,2-dien-4-ol (13)

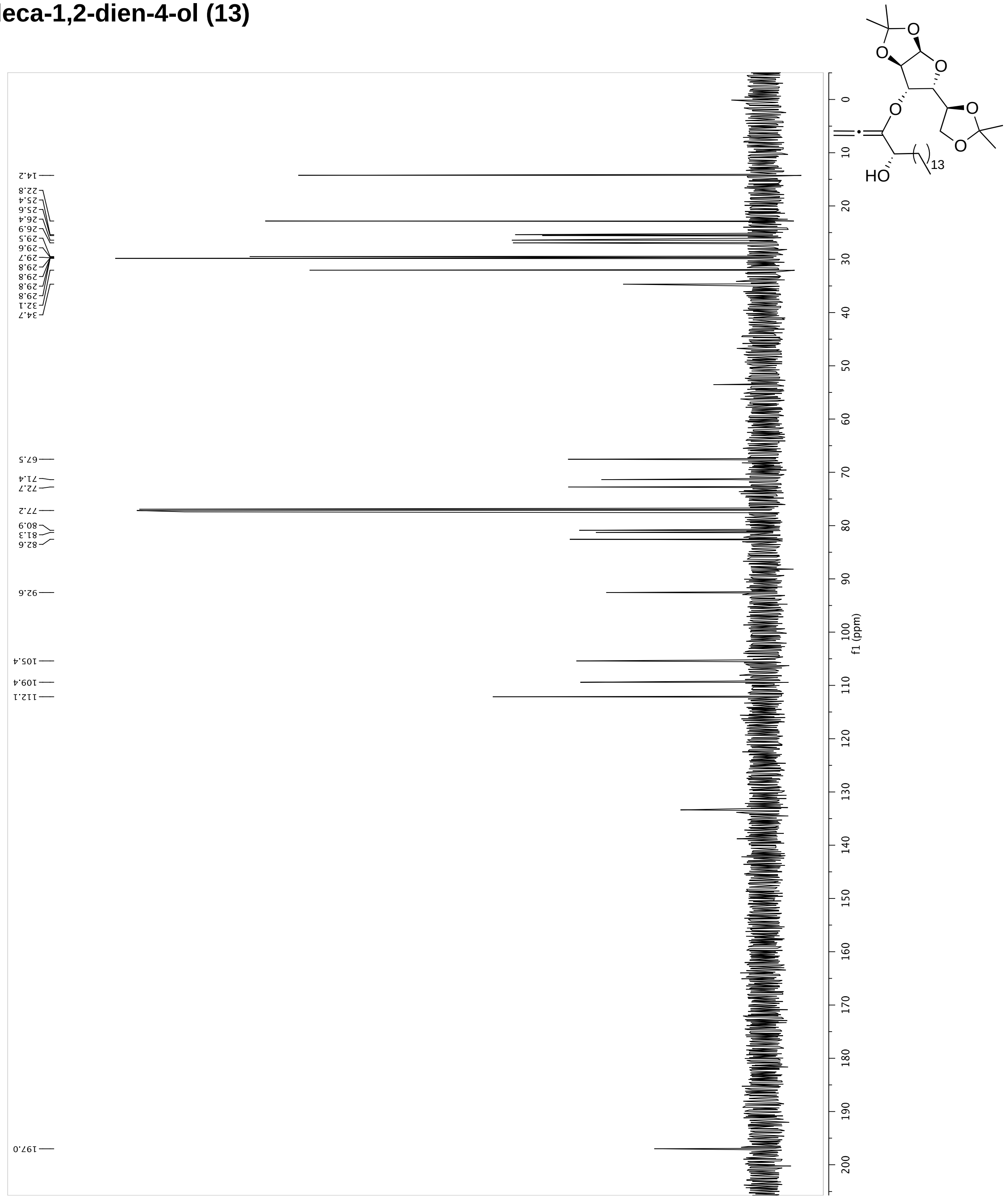

^{1}H NMR (500 MHz, $CDCl_3$) (3a*R*,5*R*,6*S*,6a*R*)-5-[(*S*)-2,2-Dimethyl-1,3-dioxolan-4-yl]-2,2-dimethyl-6-[(2-tetra-decyl-2,5-dihydrofuran-3-yl)oxy]tetrahydro-furo[2,3-*d*][1,3]dioxole (14)

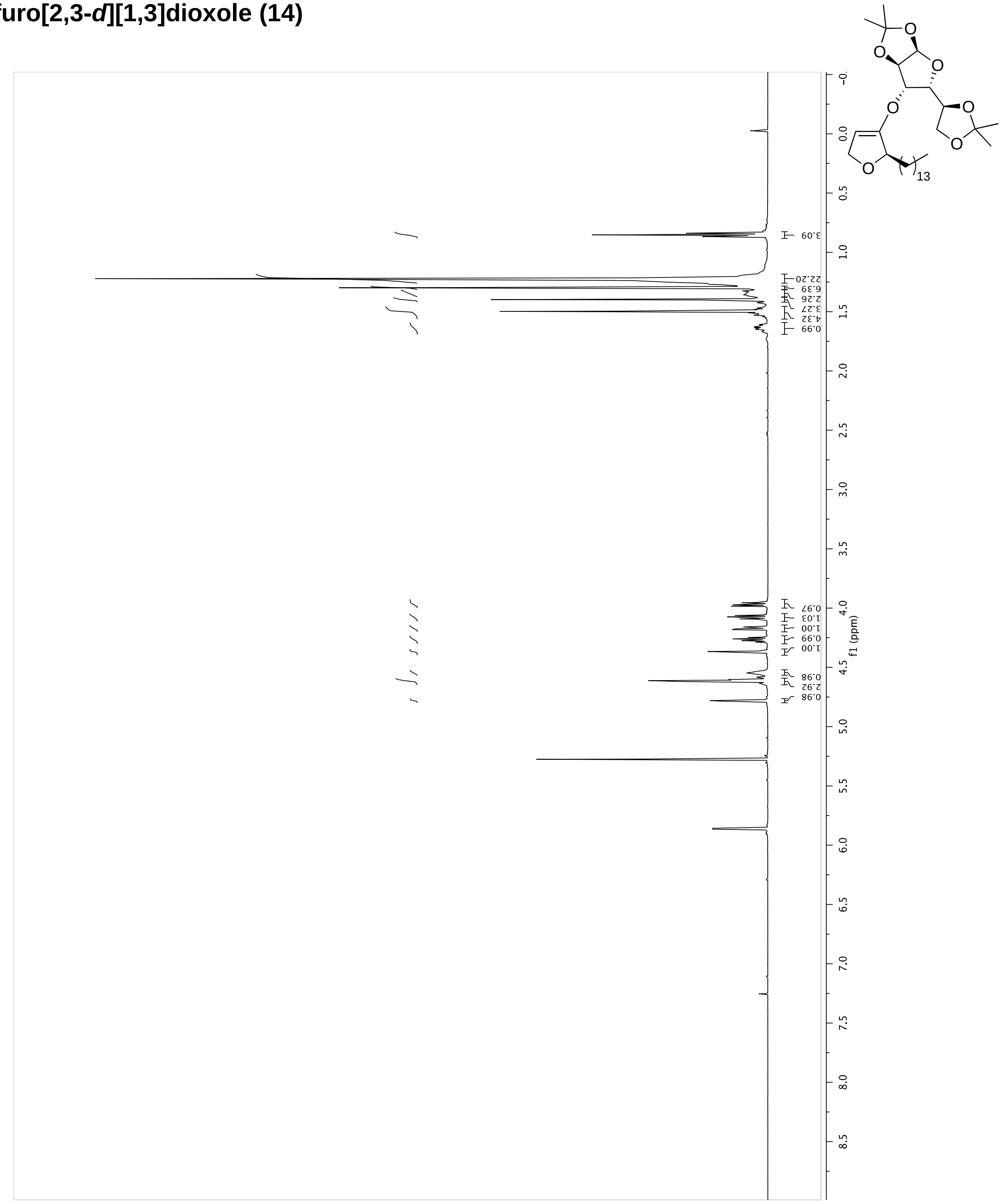

^{13}C NMR (126 MHz, $CDCl_3$) (3a*R*,5*R*,6*S*,6a*R*)-5-[(*S*)-2,2-Dimethyl-1,3-dioxolan-4-yl]-2,2-dimethyl-6-[(2-tetra-decyl-2,5-dihydrofuran-3-yl)oxy]tetrahydro-furo[2,3-*d*][1,3]dioxole (14)

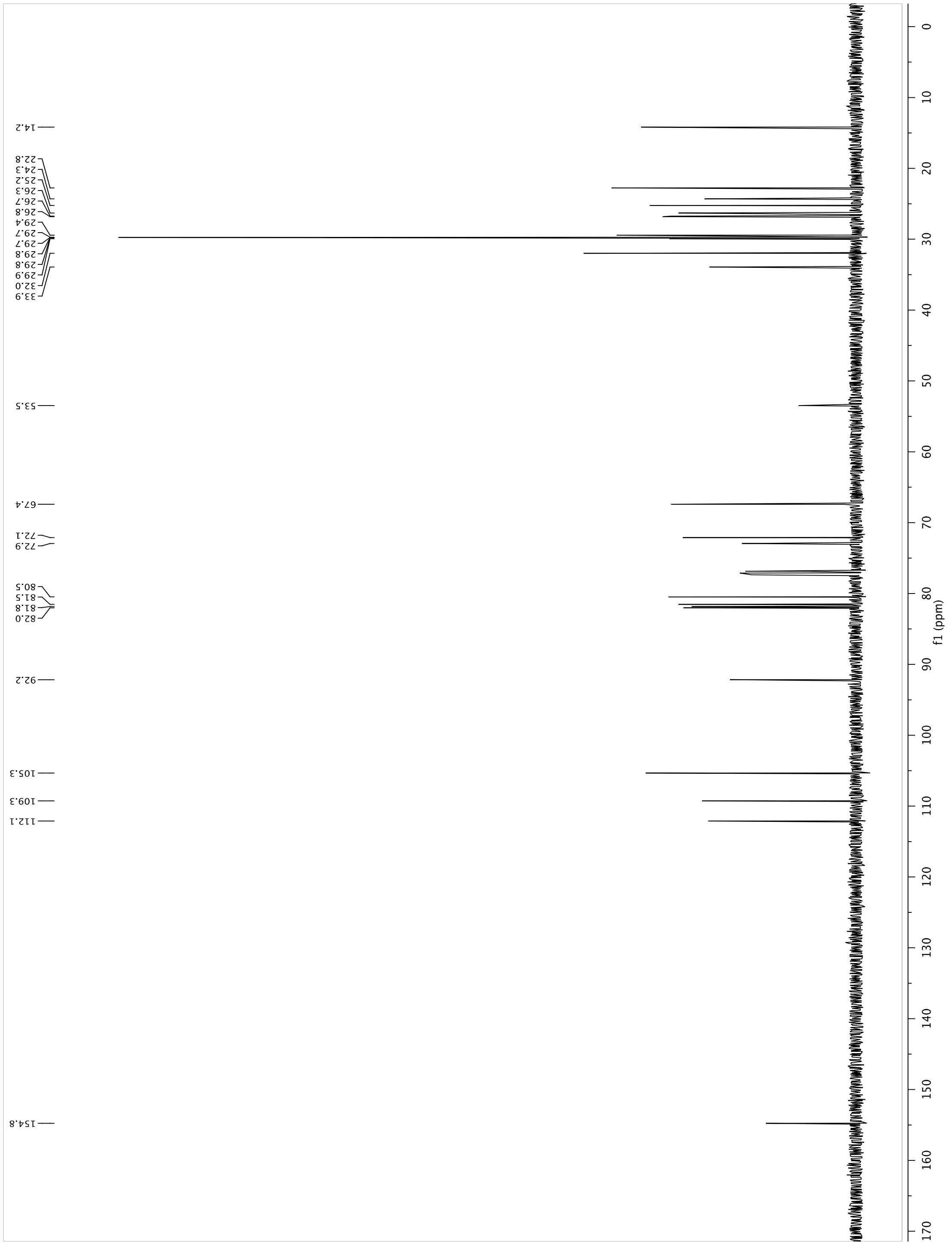

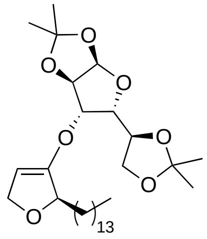

^{1}H NMR (500 MHz, CDCl$_3$) (3a*R*,5*R*,6*S*,6a*R*)-5-[(*S*)-2,2-Dimethyl-1,3-dioxolan-4-yl]-2,2-dimethyl-6-[(2-tetra-decyl-2,5-dihydrofuran-3-yl)oxy]tetrahydro-furo[2,3-*d*][1,3]dioxole (15)

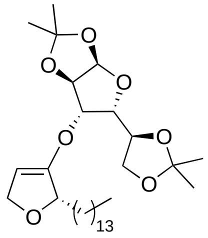

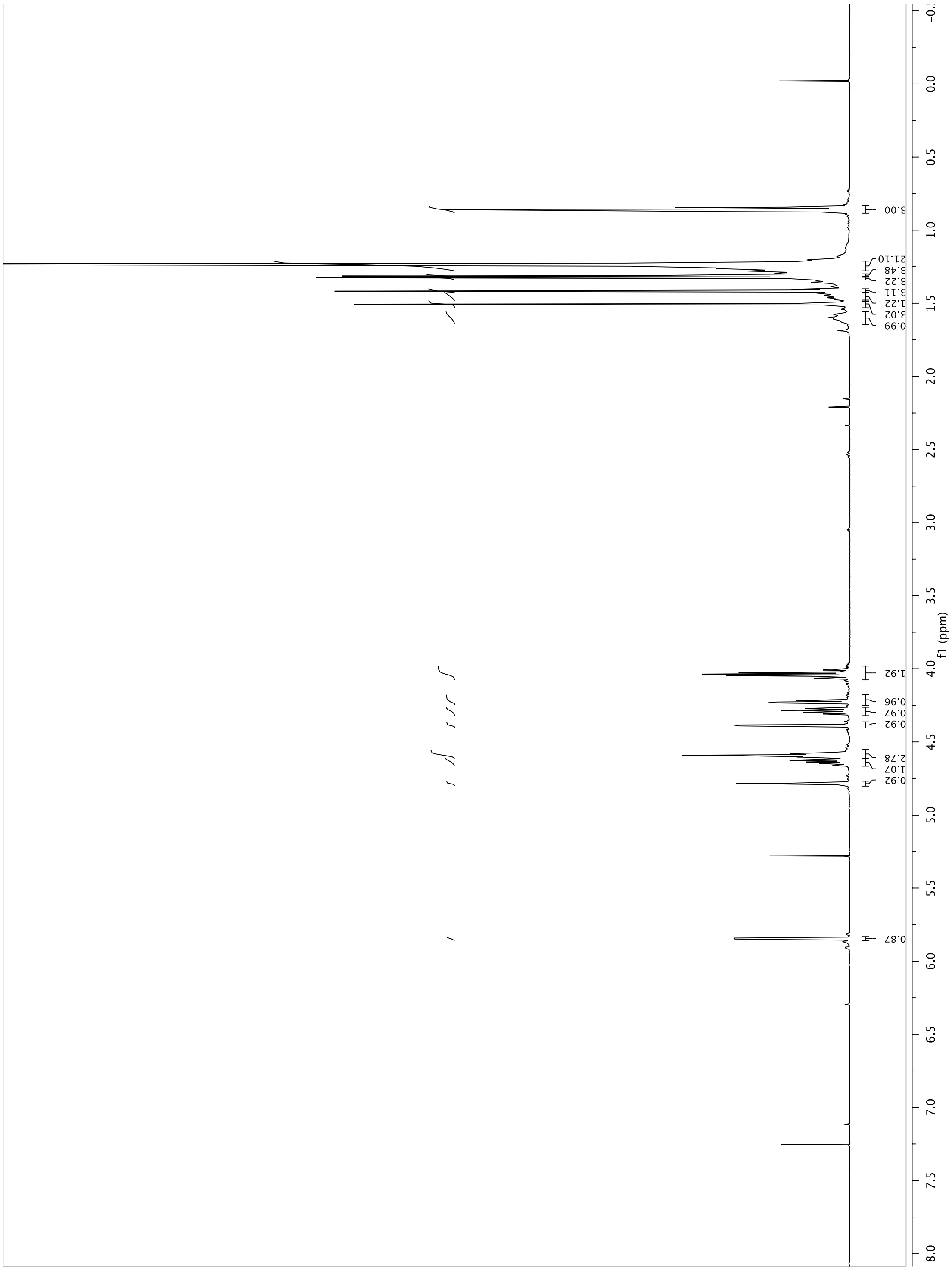

^{13}C NMR (126 MHz, $CDCl_3$) (3a*R*,5*R*,6*S*,6a*R*)-5-[(*S*)-2,2-Dimethyl-1,3-dioxolan-4-yl]-2,2-dimethyl-6-[(2-tetra-decyl-2,5-dihydrofuran-3-yl)oxy]tetrahydro-furo[2,3-*d*][1,3]dioxole (15)

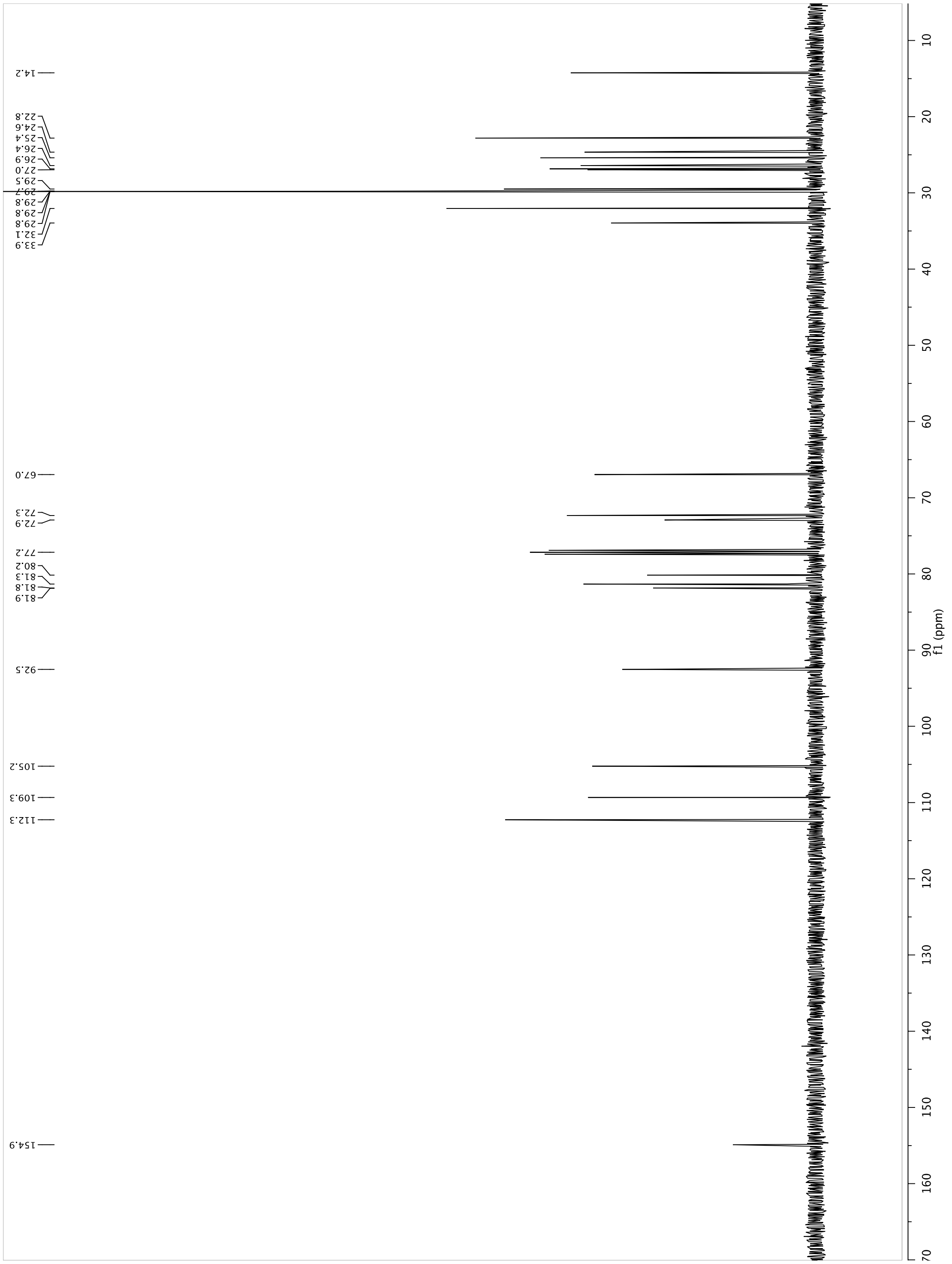

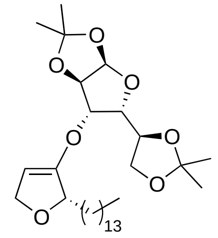

^{1}H NMR (500 MHz, CDCl$_3$) (2*S*,3*R*,4*R*)-4-Azido-2-(tetradecyl)tetrahydrofuran-3-yl benzyl carbonates (16)

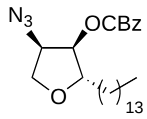

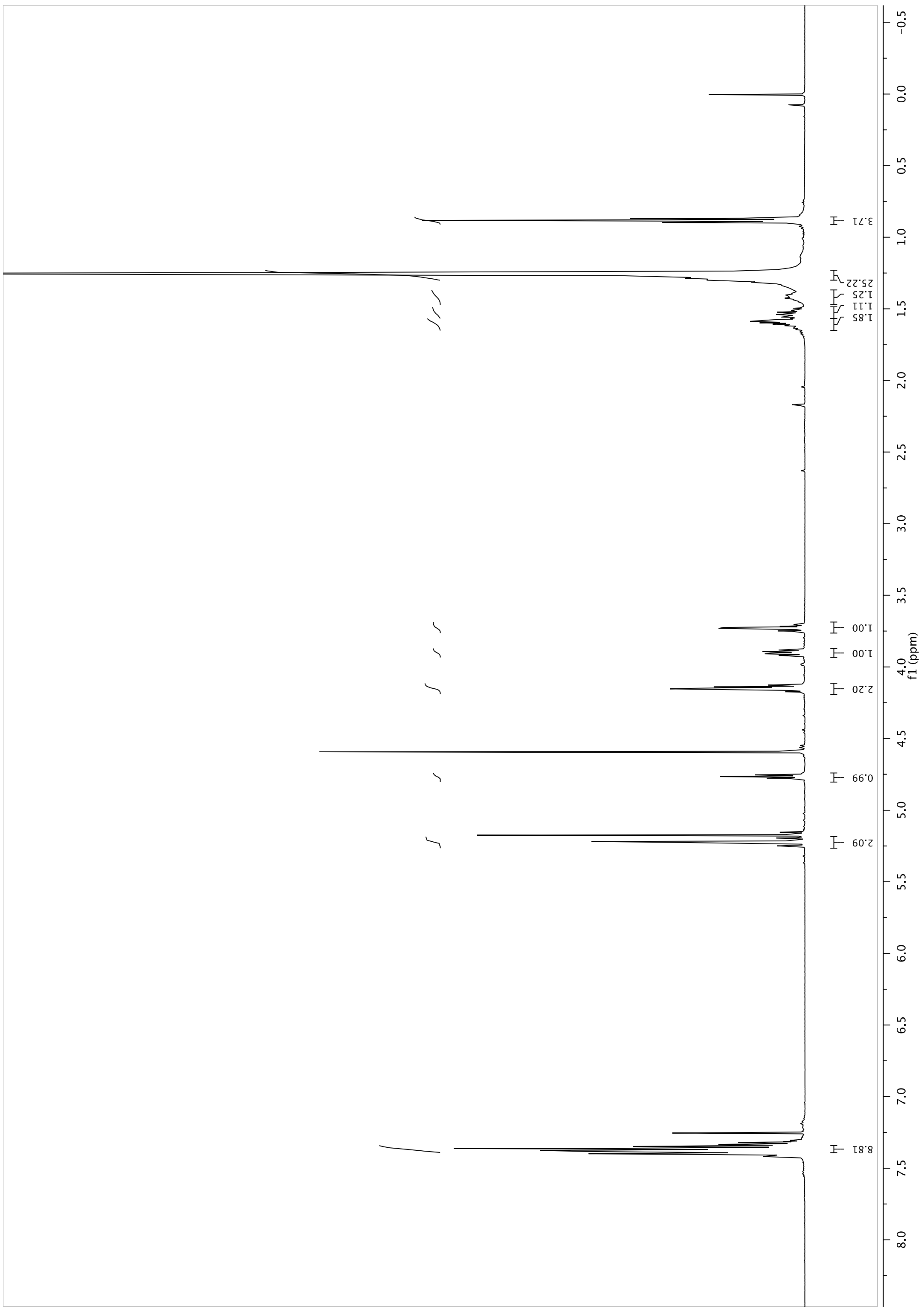

^{13}C NMR (126 MHz, CDCl$_3$) (2*S*,3*R*,4*R*)-4-Azido-2-(tetradecyl)tetrahydrofuran-3-yl benzyl carbonates (16)

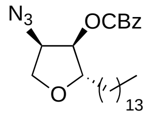

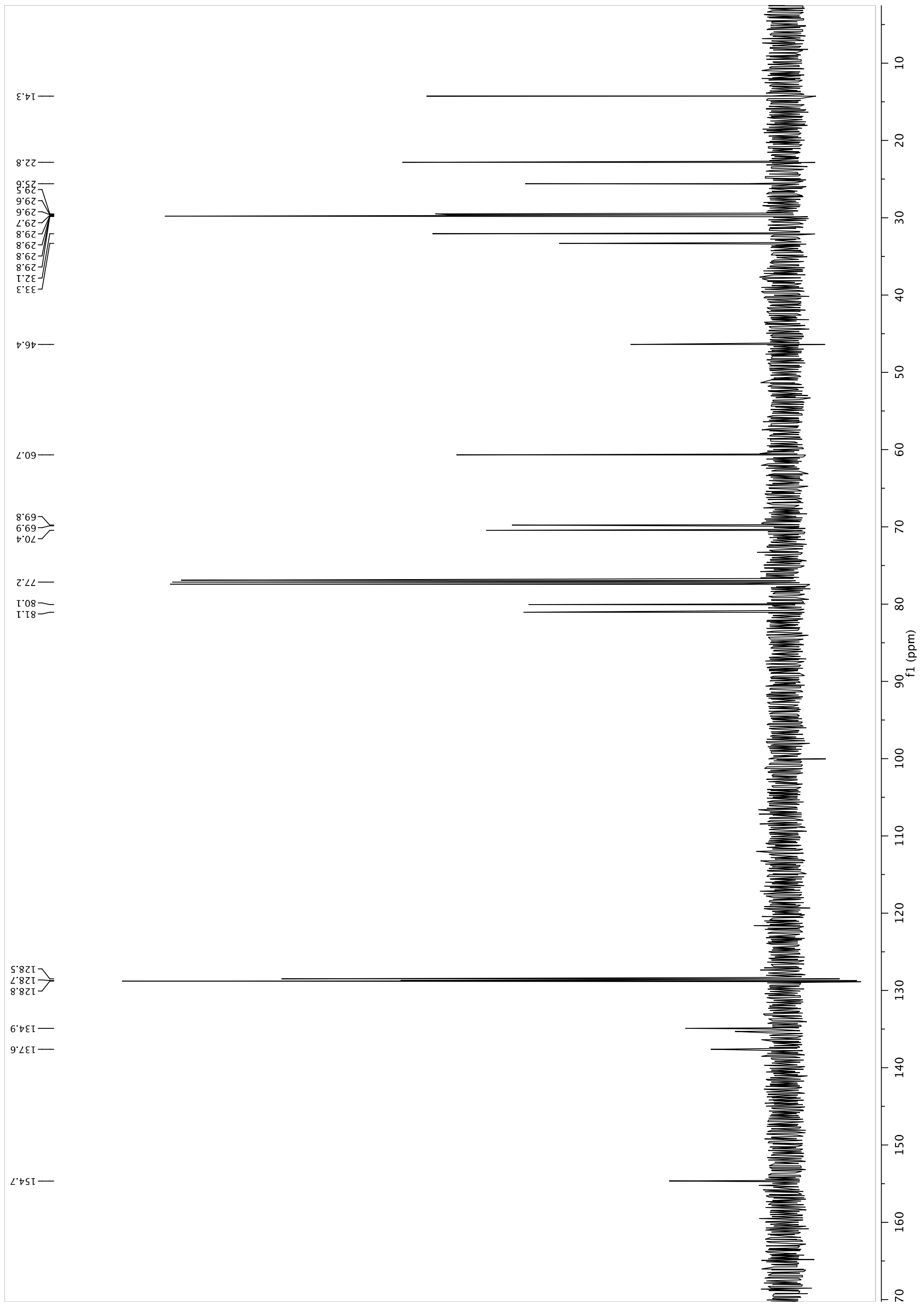

^{1}H NMR (500 MHz, $CDCl_3$) (2*S*,3*S*,4*S*)-4-Azido-2-(tetradecyl)tetrahydrofuran-3-yl benzyl carbonates (17)

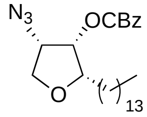

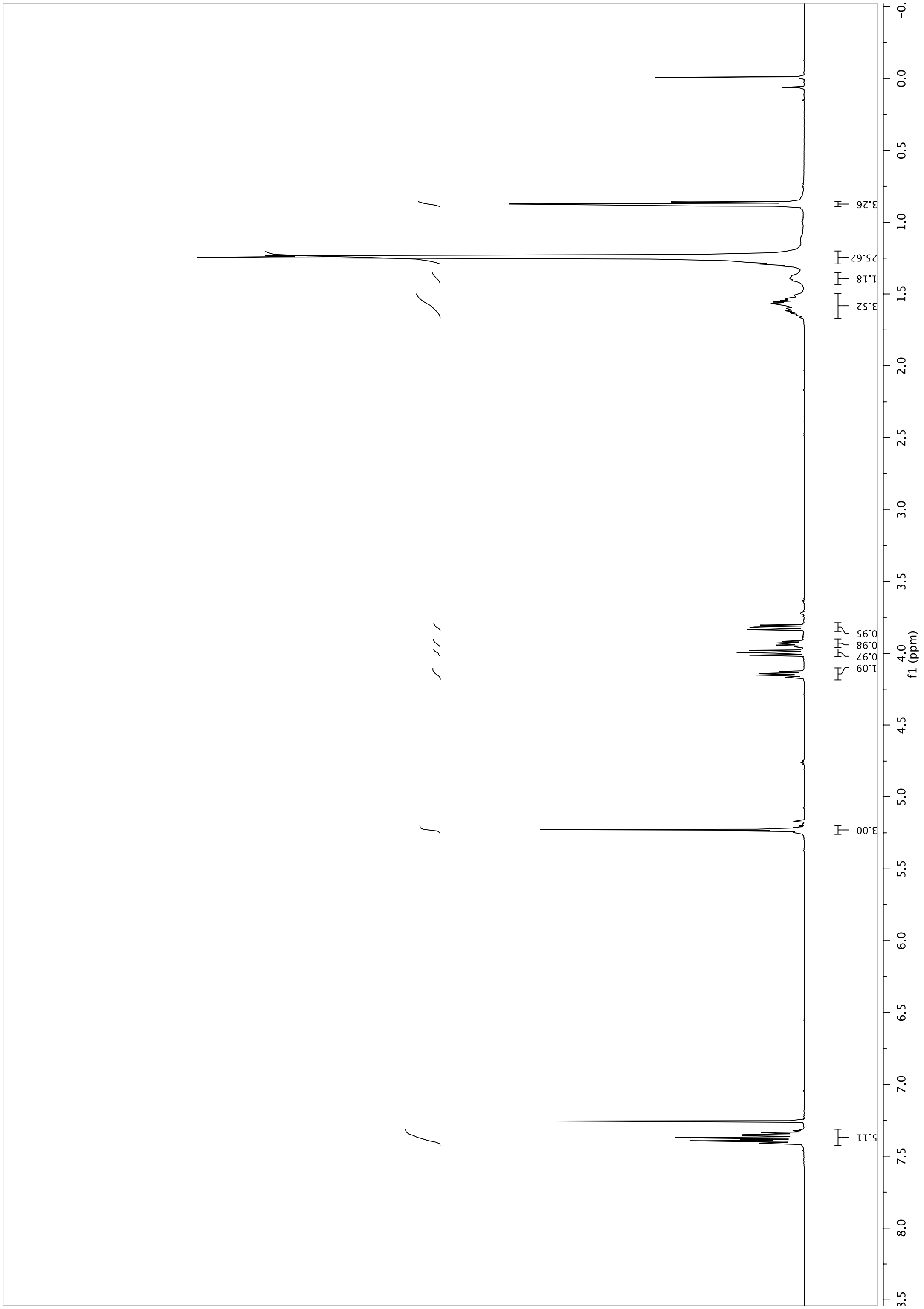

^{13}C NMR (126 MHz, CDCl$_3$) (2*S,3S,4S*)-4-Azido-2-(tetradecyl)tetrahydrofuran-3-yl benzyl carbonates (17)

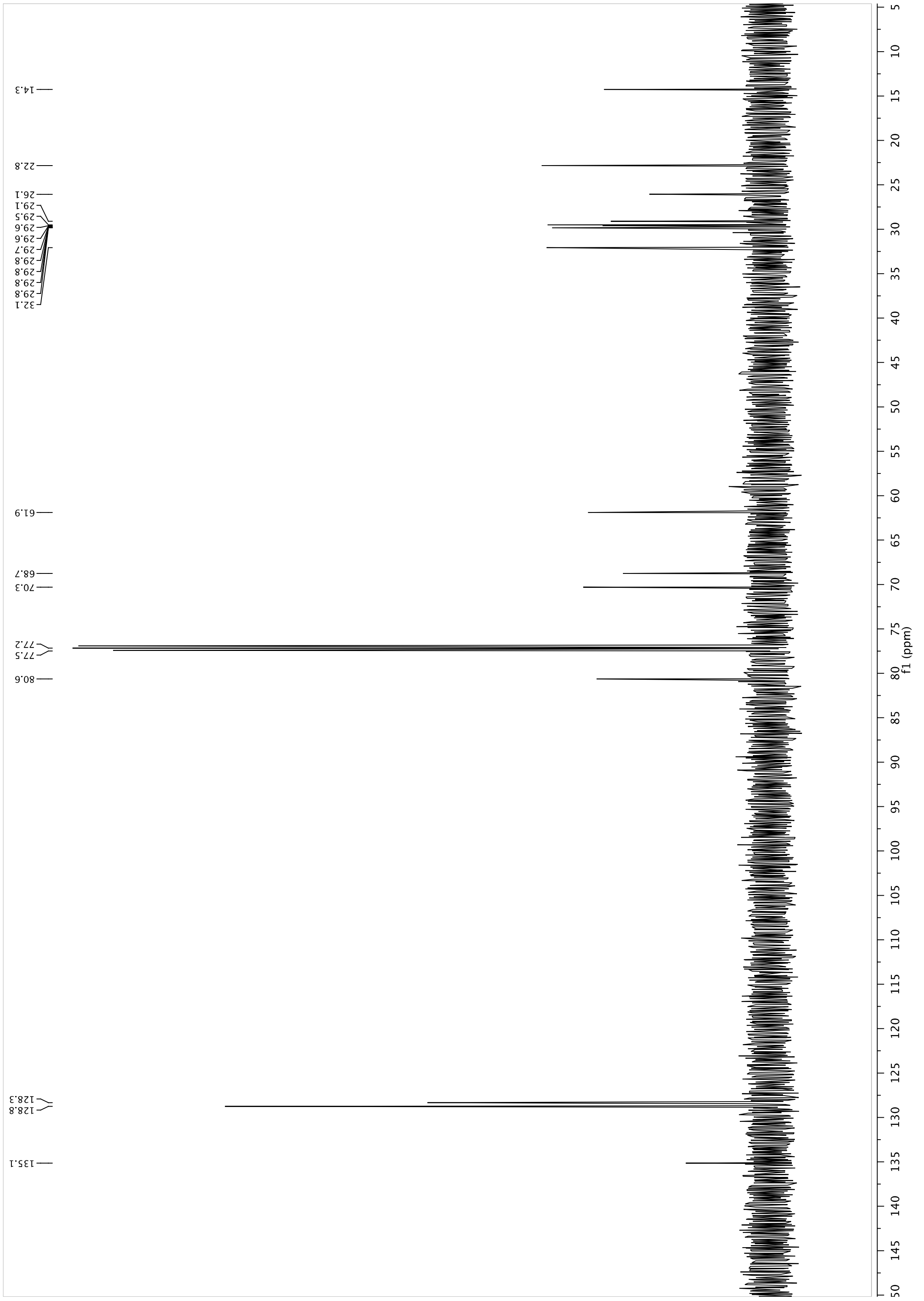

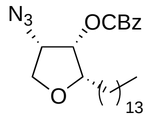

^{1}H NMR (500 MHz, CD_3OD) (2*S*,3*R*,4*R*)-4-Amino-2-(tetradecyl)tetrahydrofuran-3-ol (2)

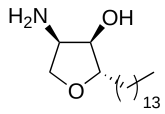

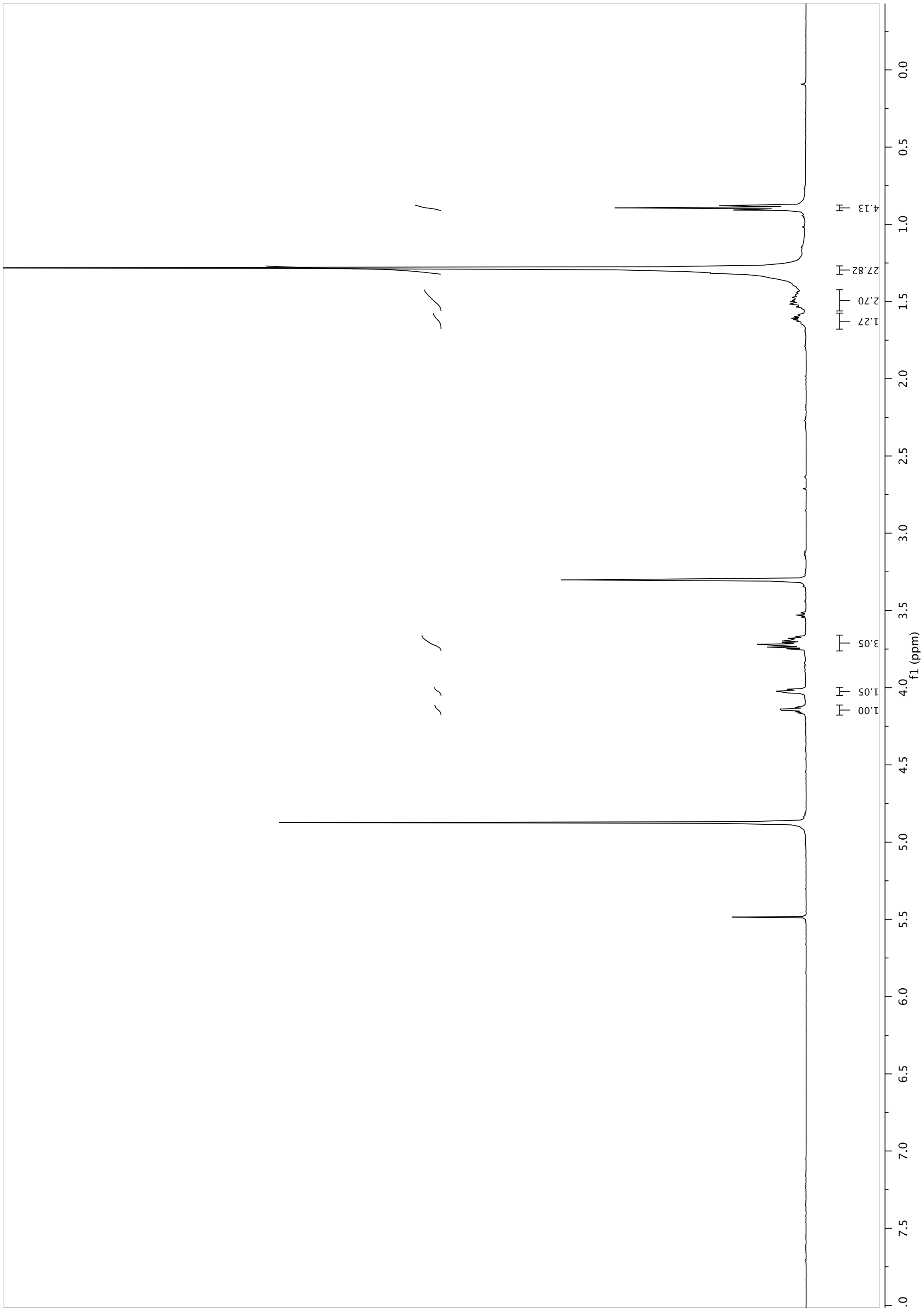

^{13}C NMR (126 MHz, CD_3OD) (2*S*,3*R*,4*R*)-4-Amino-2-(tetradecyl)tetrahydro-furan-3-ol (2)

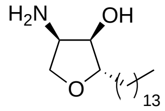

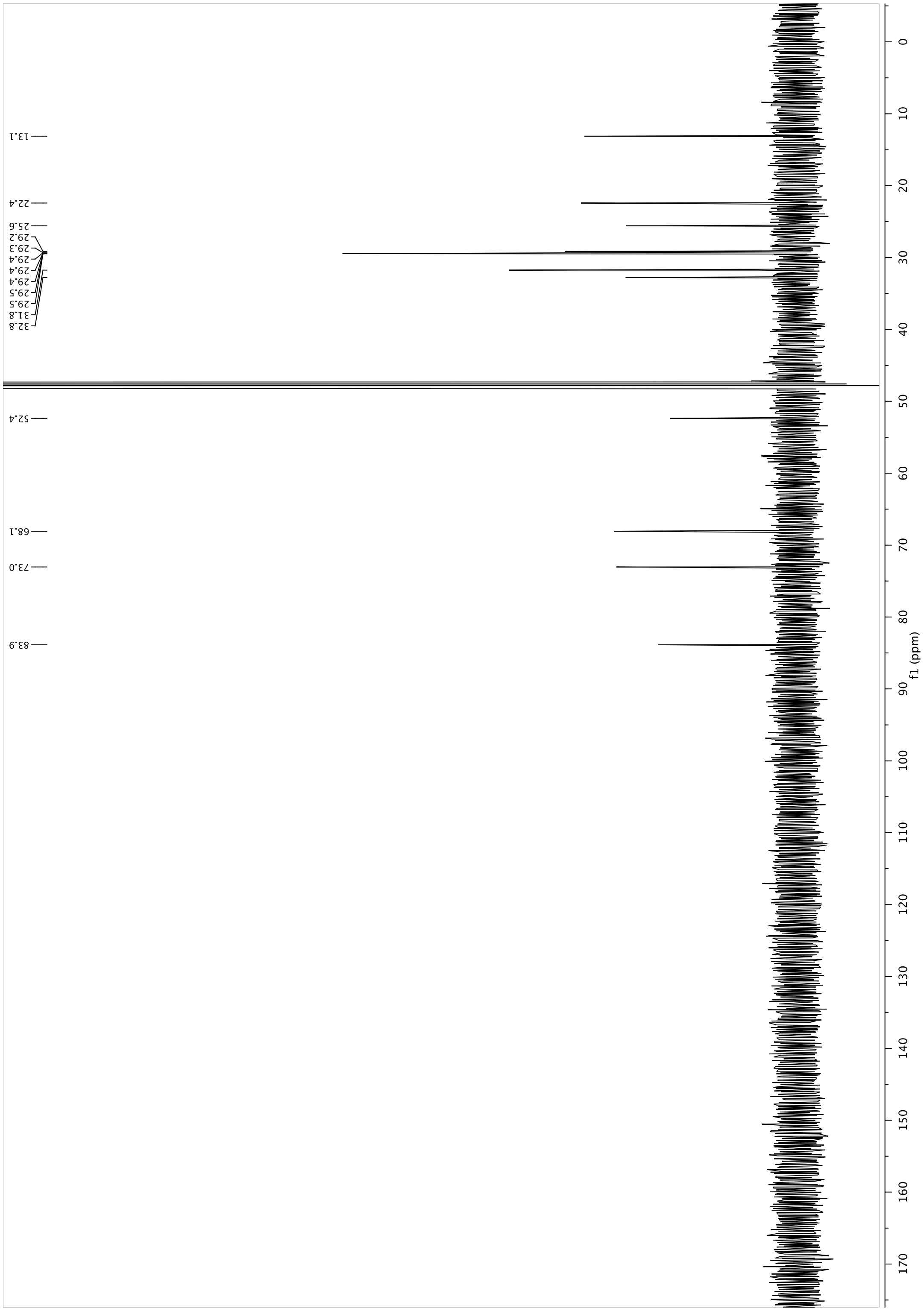

^{1}H NMR (500 MHz, $CDCl_3$) Jaspine B 1

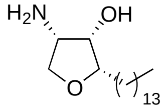

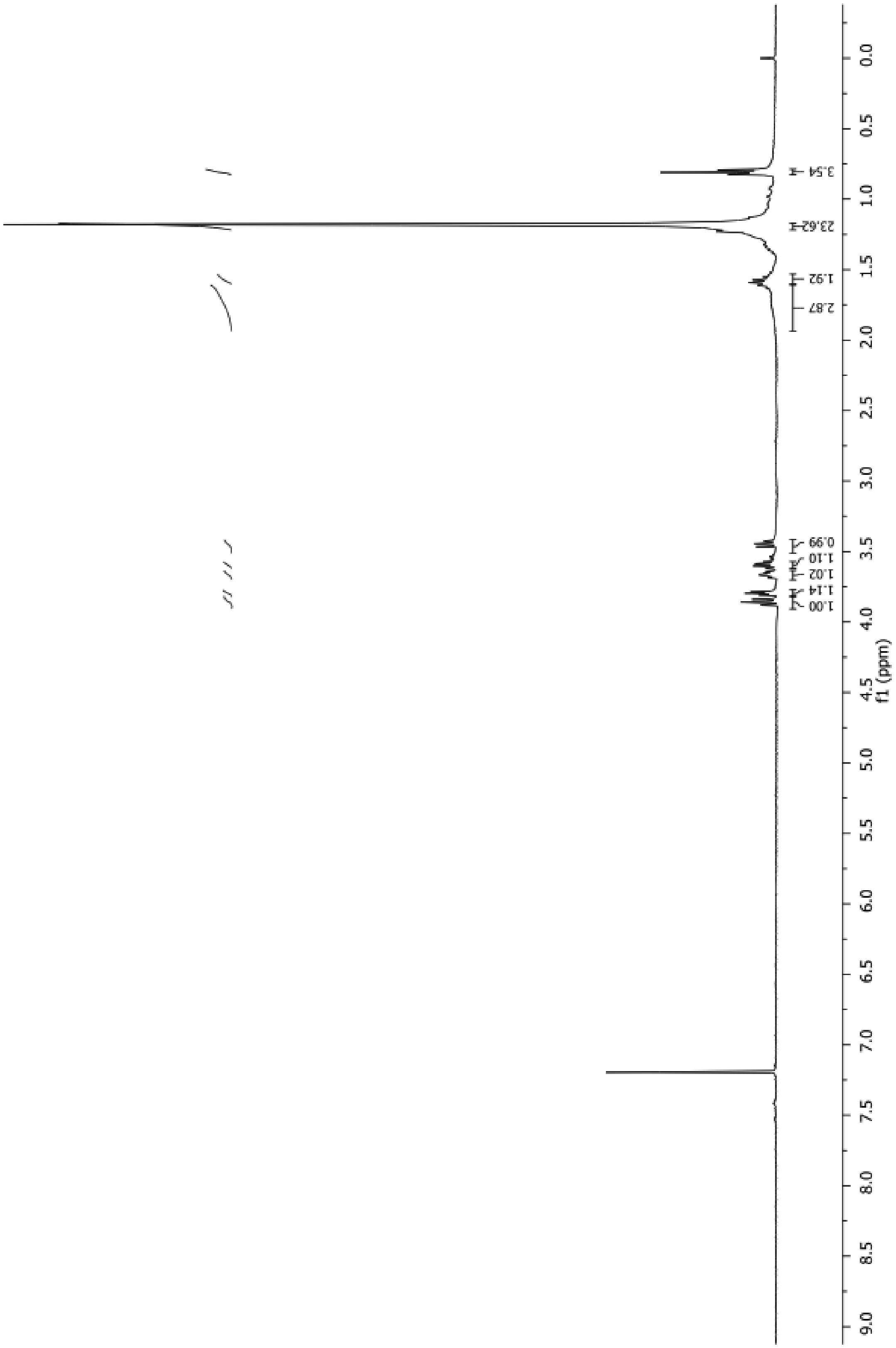

^{13}C NMR (126 MHz, $CDCl_3$) Jaspine B 1

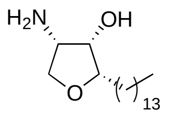

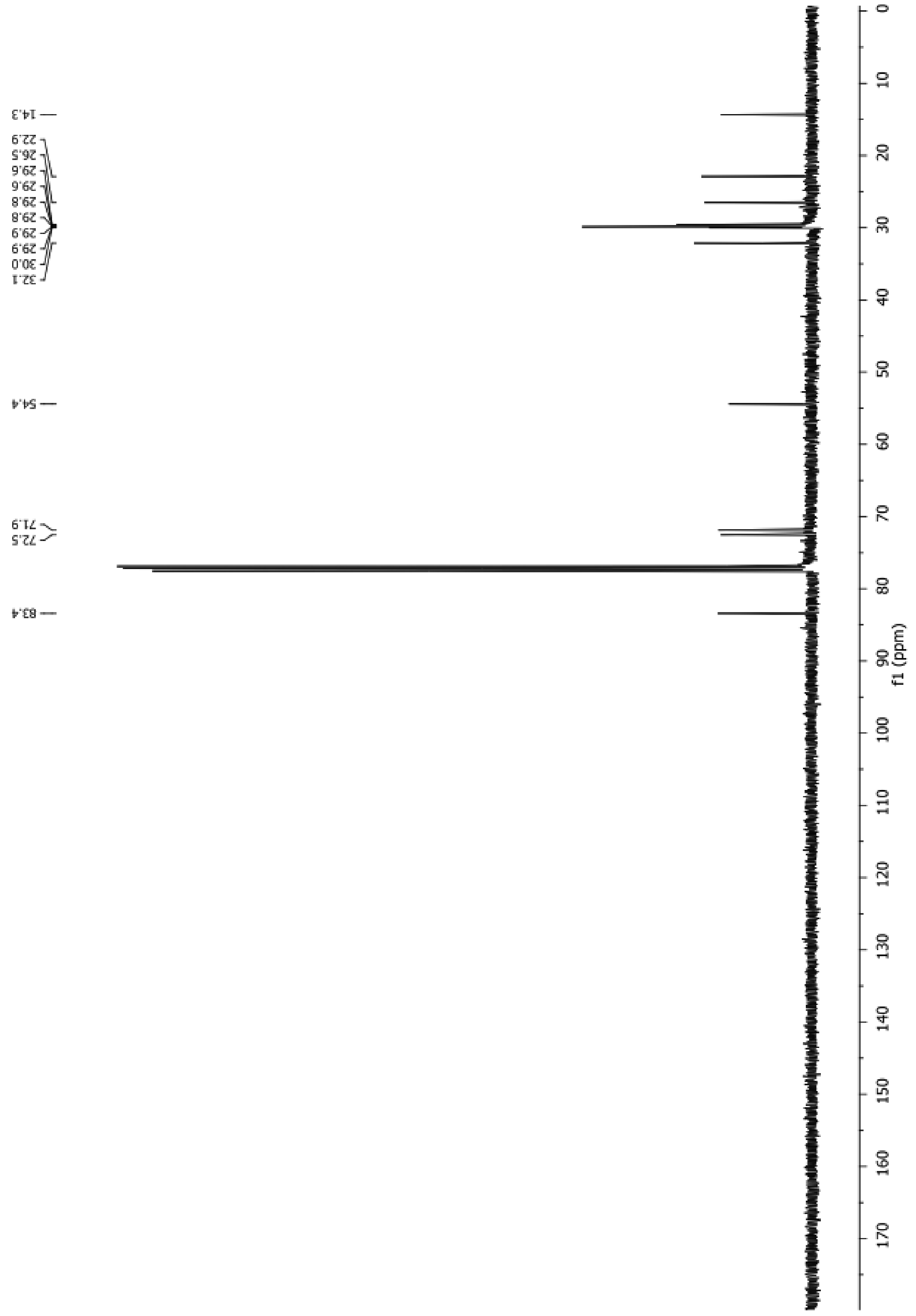

Supplement: File 1 — Experimental procedures and analytical data and NMR spectra. [file Beilstein_J_Org_Chem-09-2564-s001.pdf]
